# Investigation of the effects of phthalates on *in vitro* thyroid models with RNA-Seq and ATAC-Seq

**DOI:** 10.3389/fendo.2023.1200211

**Published:** 2023-09-22

**Authors:** Marta Nazzari, Mírian Romitti, Duncan Hauser, Daniel J. Carvalho, Stefan Giselbrecht, Lorenzo Moroni, Sabine Costagliola, Florian Caiment

**Affiliations:** ^1^ Department of Toxicogenomics, GROW School for Oncology and Reproduction, Maastricht University, Maastricht, Netherlands; ^2^ Institute of Interdisciplinary Research in Molecular Human Biology (IRIBHM), Université Libre de Bruxelles, Brussels, Belgium; ^3^ Department of Instructive Biomaterials Engineering, MERLN Institute for Technology-Inspired Regenerative Medicine, Maastricht University, Maastricht, Netherlands; ^4^ Department of Complex Tissue Regeneration, MERLN Institute for Technology-Inspired Regenerative Medicine, Maastricht University, Maastricht, Netherlands

**Keywords:** ATAC-seq, Nthy-ori 3-1, organoids, phthalates, RNA-seq, thyroid

## Abstract

**Introduction:**

Phthalates are a class of endocrine-disrupting chemicals that have been shown to negatively correlate with thyroid hormone serum levels in humans and to cause a state of hyperactivity in the thyroid. However, their mechanism of action is not well described at the molecular level.

**Methods:**

We analyzed the response of mouse thyroid organoids to the exposure to a biologically relevant dose range of the phthalates bis(2-ethylhexyl) phthalate (DEHP), di-iso-decylphthalate (DIDP), di-iso-nonylphthalate (DINP), and di-n-octylphthalate (DnOP) for 24 h and simultaneously analyzed mRNA and miRNA expression via RNA sequencing. Using the expression data, we performed differential expression and gene set enrichment analysis. We also exposed the human thyroid follicular epithelial cell line Nthy-ori 3-1 to 1 µM of DEHP or DINP for 5 days and analyzed changes in chromatin accessibility via ATAC-Seq.

**Results:**

Dose-series analysis showed how the expression of several genes increased or decreased at the highest dose tested. As expected with the low dosing scheme, the compounds induced a modest response on the transcriptome, as we identified changes in only mmu-miR-143-3p after DINP treatment and very few differentially expressed genes. No effect was observed on thyroid markers. Ing5, a component of histones H3 and H4 acetylation complexes, was consistently upregulated in three out of four conditions compared to control, and we observed a partial overlap among the genes differentially expressed by the treatments. Gene set enrichment analysis showed enrichment in the treatment samples of the fatty acid metabolism pathway and in the control of pathways related to the receptor signalling and extracellular matrix organization. ATAC-Seq analysis showed a general increase in accessibility compared to the control, however we did not identify significant changes in accessibility in the identified regions.

**Discussion:**

With this work, we showed that despite having only a few differentially expressed genes, diverse analysis methods could be applied to retrieve relevant information on phthalates, showing the potential of in vitro thyroid-relevant systems for the analysis of endocrine disruptors.

## 1 Introduction

Phthalates are a class of manmade compounds used in the manufacturing industry as solvents or added as plasticizers, mainly polyvinyl chloride (PVC) or other polymers, to confer flexibility and softness (1, 2). Phthalates are alkyl or dialkyl esters of phthalic acid, and their functional groups can be linear, branched, or circular (3). Depending on their size, phthalates are classified into low and high molecular weights (MW) (4, 5). Low-MW phthalates include benzyl butyl phthalate (BBP), diethyl phthalate (DEP), di-*iso*-butyl phthalate (DiBP), dimethyl phthalate (DMP), and di-*n*-butyl phthalate (DnBP), while high-MW ones comprise *bis*(2-ethylhexyl) phthalate (DEHP), di(2-propylheptyl) phthalate (DPHP), di-*iso*-decylphthalate (DIDP), di-*iso*-nonylphthalate (DINP), and di-*n*-octylphthalate (DnOP) (6). They are found in common household items, medical devices, construction materials, and consumer products (6). Since they are not covalently bound to the plastic matrix they are contained in, phthalates can leach or gas out and contaminate either the environment or be ingested via contaminated food (5). Indeed, food constitutes one of the biggest sources of human exposure to phthalates (7). Human biomonitoring studies conducted on the general population in Asia, Europe, and North America show a widespread exposure of the general population to phthalates (6, 8–11). Despite their broad use and pervasive environmental presence, they have been recognized as toxic substances both in humans and other organisms (12). Once ingested, they are rapidly metabolized in the digestive tract to their monoester form, which is the species responsible for the phthalates’ toxicity. Low-MW phthalate metabolites are then excreted through the urine, while high MW metabolites are excreted both via the urine and feces (1). While they do not bioaccumulate, their persistent exposure of the population is a matter for concern. Short- and medium-chain phthalates have been associated with higher toxicity than long-chain ones, which has led to their banning or restriction in children’s toys or teething products (13, 14). Some of the examined compounds have been reported to cause chronic or subchronic toxicity in several organs and systems, namely the liver, kidney, immune system, testes, uterus, ovary, central nervous system, and thyroid *in vivo* (2, 15–17). Phthalates can also negatively interfere with the endocrine system and are thus considered endocrine-disrupting chemicals (EDCs). They have been shown to interfere with prenatal and postnatal development in animal models (18), with the female and male reproductive systems (19–21), as well as being possibly linked to obesity and type 2 diabetes (22–24).

The thyroid is an endocrine gland positioned in the lower part of the anterior neck and is responsible for the production of the thyroid hormone (TH), whose receptors are expressed throughout the body (25). The TH is essential for normal growth and development and metabolism regulation (26). Its production is mainly regulated by the thyroid-stimulating hormone (TSH), which is secreted by the adenohypophysis. In turn, TSH production is regulated both by circulating TH levels and the thyrotropin-releasing hormone (TRH), synthesized in the hypothalamus. The main cell type of the thyroid is constituted by thyrocytes, which organize in small hollow spheres called follicles and are responsible for synthesizing the TH, which is stored in the center of the follicle (the lumen) in a dense matrix termed colloid. The synthesis of the TH starts with the active transport of iodine inside the thyrocyte via the sodium iodide symporter (NIS in humans). In the follicle lumen, it is covalently bound via oxidation to the tyrosyl (Tyr) residues of the protein thyroglobulin (TG) via the action of the membrane-bound enzyme thyroid peroxidase (TPO) (27, 28). Following TSH stimulation, TG is degraded in the lysosomes, freeing TH, which can be transported outside the thyrocyte.

In the thyroid, phthalate treatment has been shown to have an effect *in vitro* and *in vivo*, causing histological changes such as reduced follicle size and colloid density, hypertrophy of the Golgi apparatus, an increase in the number and size of lysosomes, and alteration of the TH levels (2, 15, 16, 29, 30). DEHP has been shown to downregulate *Tshr* (Tsh receptor) expression and interfere with the Tsh/Tshr signaling pathway *in vivo* (31, 32). In humans, the presence of phthalate metabolites in urine has been observed in association with alterations in TH and TSH serum levels (22, 29). In addition, there is evidence for phthalates altering the methylation status in sperm cells (33) and adrenal glands (34) of the offspring of exposed rats, as well as the expression or activity of histone deacetylases and histone methyltransferases (35, 36).

Over the years, great effort has been made to develop thyroid organoids using both embryonic and induced pluripotent stem cells (37) that can be used for developing thyroid disease models (38) and performing cancer (39) and toxicological and drug screening (40). In the context of toxicology, *in vitro* models can offer high throughput capability, and mechanistic insight into endocrine disruption and reduce the use of animal testing, in line with the 3Rs principles for animal welfare (replacement, reduction, and refinement) (41).

In this work, we analyzed our ability to identify alterations induced by phthalate treatment by using two *in vitro* models of the thyroid. To this end, we exposed mouse embryonic stem cell (mESC)-derived thyroid follicles (42) to the high-MW phthalates DEHP, DIDP, DINP, and DnOP for 24 h and analyzed the transcriptome via RNA-Sequencing (RNA-Seq) using the Combo-Seq library prep kit for simultaneous analysis of mRNA and miRNA expression. Data analysis revealed the upregulation of the growth protein 5 (*Ing5*) gene in three out of four tested compounds (DEHP, DINP, DnOP) compared to the control. ING5 is a component of the histones H3 and H4 acetyltransferase complexes HBO1-JADE, HBO1-BRPF1, and MOZ/MORF (43, 44). To investigate the potential effect of phthalate treatment on the chromatin status of thyroid models *in vitro*, we exposed the human thyroid follicular epithelial cell line Nthy-ori 3-1 to 1 µM of DEHP or DINP for 5 days and analyzed the genome accessibility with *Assay for Transposase-Accessible Chromatin* (ATAC)-Seq. We used maSigPro to analyze gene expression across the dose series and performed gene set enrichment analysis (GSEA) to identify enriched pathways.

## 2 Materials and methods

### 2.1 Chemicals information

The following phthalates were used for the experiments described in this paper: DEHP (CAS 117-81-7; purity: 99.8% ± 0.4%) (67261, Sigma-Aldrich, St. Louis, MO, USA), DINP (CAS 28553-12-0; ester content: ≥ 99% mixture of C_9_ isomers) (376663, Sigma-Aldrich), DIDP (CAS 26761-40-0; purity: ≥ 99.0%) (80135, Supelco, St. Louis, MO, USA), and DnOP (CAS 117-84-0; purity ≥ 99.5%) (D201154, Sigma-Aldrich).

### 2.2 Organoids differentiation

Thyroid organoids were differentiated from the A2Lox.Cre_TRE-Nkx2-1/Pax8_Tg-EGFP mouse ESC and enriched as previously described (42, 45). For more information, see **Supplementary Methods**.

### 2.3 Exposure to phthalates and RNA-Seq library preparation

#### 2.3.1 Exposure to phthalates

In low-adhesion 48-well cell culture plates, 1,000 follicles per well were seeded in triplicate using supplemented differentiation medium (**Supplementary Methods**) and 1–10–100 nM to 1–10 μM of DEHP, DINP, or DIDP or 2–20–200 nM to 2–20 μM of DnOP dissolved in DMSO (1029521000, Merck Millipore, Burlington, MA, USA) (final DMSO concentration: 0.5%). Of note, the slightly different dose range for DnOP was caused by an unwanted dilution error. We decided to still consider DnOP not differently than the other three phthalates in our following data analysis, considering the dose range still maintains the same scaling between each dose and its order of magnitude is comparable to the others. As a control, 1,000 follicles per well were seeded in a supplemented differentiation medium and 0.5% DMSO (*n* = 5) or medium alone (*n* = 3). The plated follicles were incubated at 37°C, 5% CO_2_, and > 95% humidity for 24 h.

#### 2.3.2 RNA isolation

After 24 h, the follicles were collected, washed once with PBS, and lysed in QIAzol Lysis Reagent (79306, Qiagen, Venlo, The Netherlands). Total RNA was extracted using the miRNAeasy Micro Kit (217084, Qiagen). All samples had a RNA integrity number (RIN) of 8 or higher.

#### 2.3.3 RNA-Seq library preparation

The NEXTFLEX® Combo-Seq™ mRNA/miRNA Kit (NOVA-5139-53, PerkinElmer, Waltham, MA, USA) was used to prepare RNA-Seq libraries using 20 ng of total RNA. To deplete tRNA and Y RNA fragments, the NEXTFLEX® tRNA/YRNA Blocker was used during the library preparation following the manufacturer’s instructions. In total, 14 cycles of PCR were performed during the protocol. For some samples, the final library concentration was below the pooling concentration used for sequencing (1.6 nM). In these cases, the library was prepared again to perform 16 cycles. For three samples (10 μM of DEHP: replicate 3; 1 nM of DIDP: replicate 3; untreated control: replicate 1), there was not enough RNA to repeat the library preparation and could thus not be sequenced. The prepared libraries were sequenced on an S4 Illumina flowcell 35 cycles (v1.5) (Illumina) in single-end mode.

### 2.4 Exposures to DEHP or DINP and ATAC-Seq library preparation

#### 2.4.1 Exposure to DEHP or DINP

The human thyroid follicular epithelial cell line Nthy-ori 3-1 was plated at a density of 10,000 cells/cm^2^ on six-well plates and cultured in RPMI 1640 Medium with GlutaMAX™ Supplement (61870036, Gibco, Waltham, MA, USA), 10% FBS, and penicillin–streptomycin (15140122, Gibco) and incubated at 37°C, 5% CO_2_, and > 95% humidity. Cells were left 1 day to adhere, and the following day, the medium was changed to culture medium with DEHP (*n* = 6) or DINP (*n* = 6) at 1 μM in 0.5% DMSO. As solvent control, the culture medium was added with just 0.5% DMSO (*n* = 6). Cells were incubated for 5 days, refreshing the media with the compound or DMSO only at day 3. At the end of the incubation period, cells were collected and counted manually.

#### 2.4.2 ATAC-Seq libraries preparation

To prepare ATAC-Seq libraries, 50,000 cells per sample were used. Libraries were prepared following the Omni-Atac protocol of Corces et al. (46) with the replacement of NP40 from the original protocol with IGEPAL (I8896: 50 ml, Merck Millipore). The tegmental kit used was the Illumina Tagment DNA Enzyme and Buffer Small Kit (20034197, Illumina, San Diego, CA, USA) and the indexes IDT® for Illumina® DNA/RNA UD Indexes Set A, Tagmentation (96 indexes, 96 samples) (20027213, Illumina). Seven PCR cycles were used for all samples. The prepared libraries were sequenced on an SP Illumina Flowcell v1.5 (100 cycles) (Illumina) in paired-end mode.

All RNA samples and sequencing library concentrations were measured with the Qubit 2.0 Fluorometer (Thermo Fisher Scientific, Waltham, MA, USA), and quality control was performed on a BioAnalyzer 2100 expert (Agilent, Santa Clara, CA, USA) or a 2200 TapeStation System (Agilent).

### 2.5 Data analysis

All the scripts used for RNA-Seq and ATAC-Seq data analysis have been collected in a markdown file available at https://github.com/marta-nazzari/phthalates_rnaseq_atacseq.

#### 2.5.1 RNA-Seq data processing

The.fastq files were processed according to our previously published CODA pipeline (47). Briefly, reads were trimmed from the 5′ 4N and 3′ 8A adapters using Cutadapt (v3.7) (48), as recommended by the manufacturer (49). To obtain gene read counts, trimmed reads were aligned to the mouse transcriptome (GRCm39 v27) and quantified using RSEM (v1.3.3) with the “–STAR” parameter (v2.7.10a), following the ENCODE3’s STAR-RSEM pipeline (50, 51). To analyze miRNAs, the trimmed files were used as input for miRge3.0 (v0.0.9) (52) using miRBase mouse annotations (v22).

#### 2.5.2 ATAC-Seq data processing

The.fastq files were preprocessed using the PEPATAC pipeline (v0.10.4) (53) using bowtie2 (v2.4.2) (54) as mapper, samtools (v1.4) (55) as deduplicator, and the included Python tool “pyadapt” as trimmer. The human genome GRCh38 v38 build was used for alignment.

#### 2.5.3 RNA-Seq sample biotype mapping and outlier identification

Quantified RNA species were mapped to their respective biotypes using the R (56) package biomaRt (57). We calculated the percentage of mapped reads per biotype and retained only those constituting at least 1% in at least one sample. Outliers for each biotype were identified per treatment group (DEHP, DIDP, DINP, DnOP, DMSO, and untreated) and calculated as being 1.5 times the interquartile range (IQR) below the 25th percentile or above the 75th percentile:


Biotype x in sample y < 25th percentile (biotype x in group z)− 1.5*IQR (biotype x in group z)

or


Biotype x in sample y >75th percentile (biotype x in group z)+ 1.5*IQR(biotype x in group z)

#### 2.5.4 MaSigPro analysis

Normalized gene counts were used for maSigPro (58) analysis according to the maSigPro user’s guide for next-generation sequencing data (59) for a single-series course experiment. We set the “tetha” (θ) value to 10 (default), the FDR to 0.05 (default), and the “degree” parameter to 3 (this corresponds to a cubic polynomial regression model). The variable “time” with values 0, 1, 2, 3, 4, and 5 was used in the model to represent the “dose” values of 0 (DMSO control), 1 nM, 10 nM, 100 nM, 1 μM, and 10 μM (or 2 nM, 20 nM, 200 nM, 2 μM, and 20 μM for DnOP).

### 2.6 Differential expression analysis

Differential gene and miRNA expression analysis was performed comparing the phthalate-treated samples to the DMSO solvent control using the R package DESeq2 (60), following a slightly modified version of the guidelines of the Omics Data Analysis Framework for regulatory application (R-ODAF) pipeline developed by our group (61, 62). Briefly, a first filtering step (“relevance threshold”) was applied to select the expressed genes/miRNAs by retaining only those whose normalized expression is ≥ 1 count per million (CPM) in at least 75% of the samples in either group (i.e., treatment versus control). To increase statistical power, all doses of a single compound were grouped together and compared to the DMSO control. The RUVg function from the RUVSeq package (63) (*k* = 2) was used on the genes/miRNAs passing the relevance threshold filter to remove unwanted variation. Differential expression analysis on the expressed genes/miRNAs was then performed setting the FDR to 0.01. The resulting differentially expressed (DE) genes/miRNAs/snoRNAs were subjected to an additional filtering step (“spurious spikes”) to identify those cases in which a very high expression value in only one replicate in a group is responsible for a certain gene/miRNA/snoRNA to be differentially expressed. To this end, the following formula was applied to every DE gene/miRNA/snoRNA for both treatment and control groups: $\frac{\text{read count gene}/{\text{miRNA}}_{i}}{\text{total read count gene}/{\text{miRNA}}_{i}{\text{ in group}}_{j}}<1.4\times {\left({\text{number of replicates in group}}_{j}\right)}^{-0.66}$ , where i refers to any gene/miRNA/snoRNA, and j refers to either the treatment or control group. The expression of such genes/miRNAs/snoRNAs was manually checked in all replicates to determine whether a gene that failed this spurious spike filter was indeed a technical artifact or could instead be biologically relevant.

### 2.7 Gene set enrichment analysis

GSEA was performed using the R package ReactomePA (v1.40.0) (64) and Reactome as a database (65) using the DESeq2 “stat” value for gene ranking. For significance, we set a *q*-value threshold of 0.05.

### 2.8 Differential accessibility analysis

The alignment files (.bam) output by the PEPATAC pipeline were shifted with the deepTools (v3.5.1) (66) utility alignmentSieve to account for the Tn5 transposase duplication at the cut site. To identify differentially accessible (DA) regions, we used a sliding window approach with the R package csaw (v1.32.0) (67) and a modified version of the script made available by Sheikh and Blais on bioRxiv (68). For quantification, we used the five-prime reads, a sliding window of 50 bp without overlap, and a minimum number of 50 counts for a window to be retained. To calculate the background, we binned the genome into 10 kb bins. To distinguish the signal from the background, we compared each region against the global background and set a fold change compared to the background to 3. The differential accessibility analysis was performed with the R package edgeR (v3.4.0) (69), and we performed batch correction using the RUVs function (*k* = 5) from the RUV-Seq package (v1.32.0) (63). As multiple testing corrections should be performed on regions and not windows (70), we merged the regions identified as “signal” that are at most 500 bp apart, reaching up to a maximum merged region width of 5 kbp, and performed multiple-testing correction using the Benjamini–Hochberg method (FDR = 0.01).

Identified differentially accessible regions were annotated with HOMER (v3.13) (71) (genome version hg38). Regulatory region annotations were retrieved from the ENCODE Candidate Cis-Regulatory Elements (cCREs) registry (72). Coverage tracks were normalized using BeCorrect (v1.1.0) (73) and visuals extracted from the Integrative Genomics Viewer (IGV) (v2.13.2) (74).

## 3 Results

### 3.1 RNA-Seq results

To investigate the effect of phthalates on the transcriptome, we generated RNA-Seq data from mESC-derived thyroid organoids exposed to four phthalates using *in vivo* relevant concentrations (1–10–100 nM to 1–10 μM DEHP, DIDP, DINP; 2–20–200 nM to 2–20 μM DnOP) for 24 h. A schematic representation of the exposure regimen is shown in **Supplementary Figure S1**. In the following paragraphs, we provide some dataset quality control (QC) metrics followed by the results of gene expression analysis.

#### 3.1.1 RNA-Seq QC and outlier identification

Combo-Seq libraries had a median of 51.8 million (M) reads per sample (min = 17.7, max = 92.4 M) (**Supplementary Figure S2A**), with a median of 97% of sequenced reads with a quality score of 30 or more (min = 96.4%, max = 97.3%) (**Supplementary Figure S2B**). The median number of reads mapped to mRNAs was 41.8 M (min = 14.3, max = 78.8 M) and to miRNAs was 1.30 M (min = 0.16, max = 2.2 M) (**Supplementary Figure S2C**). As explained in the Methods section, we performed 16 cycles for some RNA-Seq libraries to reach the required concentration for sequencing (1.6 nM) (**Supplementary Table S1**). In consequence, this increased the percentage of snoRNA-mapping reads (**Supplementary Figure S3A**). As the read count of the protein-coding genes would be underestimated during DESeq2 normalization, we removed the snoRNA-mapping reads from the main dataset and performed the analysis of snoRNA genes separately. Boxplot of mapped read distributions per gene biotype after snoRNA removal revealed that one DMSO control replicate (DMSO_ctrl_1) was a clear outlier in multiple biotypes (**Supplementary Figure S3B**). As the DMSO samples would be used as a control for all comparisons, this outlier would have had a major impact in all downstream analyses and, importantly, in the most important biotypes (“protein coding” and “miRNA” in particular”). Although other samples were flagged as outliers in other biotypes (“processed pseudogene” or “rRNA”), this was less consistent and did not warrant further samples removal.

#### 3.1.2 MaSigPro analysis

**Figure 1:**
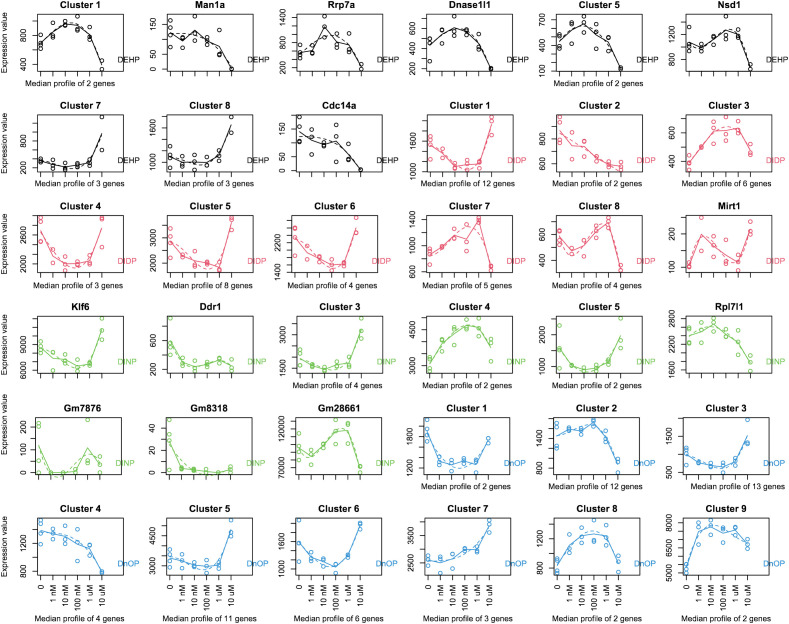
MaSigPro analysis of the gene expression over the dose series employed in the study (0–1–10–100 nM to 1–10 μM for DEHP, DIDP, and DINP and 0–2–20–200 nM to 2–20 μM for DnOP). The curves for each compound are color-coded, and the compound name is reported in every plot. Each gene was analyzed to fit up to a cubic polynomial regression model. Fits that passed multiple testing corrections (FDR = 0.05) were selected and clustered in nine groups using hierarchical clustering. If a cluster comprises only one gene, the gene name is indicated on top of the plot; otherwise, the cluster name is reported. The *x*-axis reports the dose range used (nM, nanomoles per liter; μM, micromoles per liter). The dots represent the expression values of each replicate (or the average of each gene if a cluster comprises more genes), and the dotted line shows the fit. The genes belonging to the various clusters are reported in **Supplementary Table S2**.

The MaSigPro R package, initially developed to identify changes in gene expression along a time series, can also be used to analyze the evolution of the gene expression level across a dose range exposure. Next, we investigated using MaSigPro whether some genes would show dose regulation across our six doses (untreated plus five doses). We then allowed the significant genes to be grouped into nine clusters, which include the genes that have a similar trend in change in expression over the dose series. For every compound, we observed some clusters with a nonmonotonic dose–response curve (DEHP: clusters 1 and 3–5; DIDP: clusters 1 and 3–9; DINP: clusters 1, 3–5, and 9; DnOP: clusters 1, 3, 6, and 8) (**Figure 1**). The genes belonging to the various clusters are reported in **Supplementary Table S2**. In those indicated clusters, the highest dose (10 μM or 20 μM) was consistently shown to be different from the other four. In this cellular system, this dose could be used for phthalates to derive a point of departure (PoD) metric, which in the toxicology field represents a dose at which a biological response is first observed and can be used to make extrapolations for risk assessment (75).

#### 3.1.3 Differentially expressed genes and miRNAs

Considering the divergent nature of the highest dose compared to the other four, we decided to exclude it from the differential expression analysis. Given that for each dose we had triplicates or duplicates, by consolidating all the remaining doses together and comparing them to the solvent control, we aimed at increasing the statistical power and detecting gene, miRNA, and snoRNA expression alterations specifically attributable to phthalate treatment. By doing so, we could focus on identifying changes at the compound level while accounting for the different responses observed with the highest dose.

**Figure 2:**
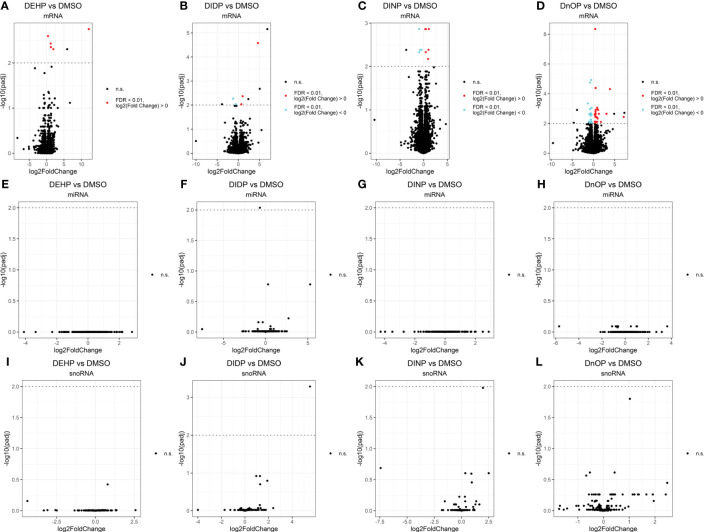
Volcano plots of the expressed genes **(A–D)**, miRNAs **(E–H)**, and snoRNAs **(I–L)** in each phthalate vs. DMSO control. Every dot represents a gene. Elements not differentially expressed (not significant – n.s.) are in black. The false discovery rate (FDR) threshold of 0.01 is indicated as a dotted line on the y-axis. Upregulated elements (“FDR < 0.01, log2(fold change) ≶ 0”) are indicated in red, and downregulated ones (“FDR < 0.01, log2(fold change)< 0”) in cyan. The genes above this line colored in black are the ones that fail to pass the “spurious spike” filter as described in the Methods section.

Differential expression analysis revealed how all the treatments had moderate effects on the cells in terms of the number of differentially expressed genes (DEGs), miRNAs, and snoRNAs (**Figure 2**): the number of DEGs compared to the control was 5, 5, 10, and 49 for DEHP, DIDP, DINP, and DnOP, respectively (FDR< 0.01) (**Table 1**). Only DIDP treatment influenced miRNA expression, with mmu-miR-143-3p being downregulated. No effect was observed on snoRNA expression or on thyroid markers (**Supplementary Figure S4**).

**Table 1:** List of differentially expressed genes (DEGs) and miRNAs (DE miRNAs) in every phthalate vs. DMSO comparison (FDR< 0.01).

	DEHP	DIDP	DINP	DnOP			
DEGs	*Gpd1*	***Plekha3***	***Acaa2***	***Acaa2***	*Hsd17b10*	***Plekha3***	*Tmem80*
	***Ing5***	*Cxcl14*	***Cops5***	*App*	*Hspa1b*	*Pole2*	*Trmt61b*
	*Myh14*	*Zgpat*	***Idh3g***	*Arhgef10l*	***Idh3g***	*Ppp1r7*	*Tspan1*
	***Acaa2***	*Rpl38-ps2*	***Ing5***	*Ccnd2*	*Ifitm3*	*Ptp4a1*	*Ube2g2*
	*Gm15516*	*Gm10323*	***Mid1***	*Ccni*	***Ing5***	***Rab5a***	*Uqcrc2*
			***Rab5a***	***Cops5***	***Mid1***	*Rars*	*Vps25*
			*Srrm1*	*Dnajc8*	*Mtpn*	*Rnf128*	*Wasf2*
			*Tnrc6b*	*Exoc6*	*Mzt1*	*Rpl19-ps11*	*Zc3h11a*
			*Ttc32*	*Fbp2*	*Npm1*	*Shroom3*	*Zfand1*
			***Zfp960***	*Gm21293*	*Nrip1*	*Sox4*	*Zfp330*
				*Gm5641*	*Nucb2*	*Tjp1*	***Zfp960***
				*Hbb-bs*	*Phlda1*	*Tmed7*	*Zswim6*
				*Herpud2*			
DE miRNAs			mmu-miR-143-3p				

Downregulated genes are reported in blue text, and upregulated ones in red. DEGs that appear in more than one comparison are in bold.

**Figure 3:**
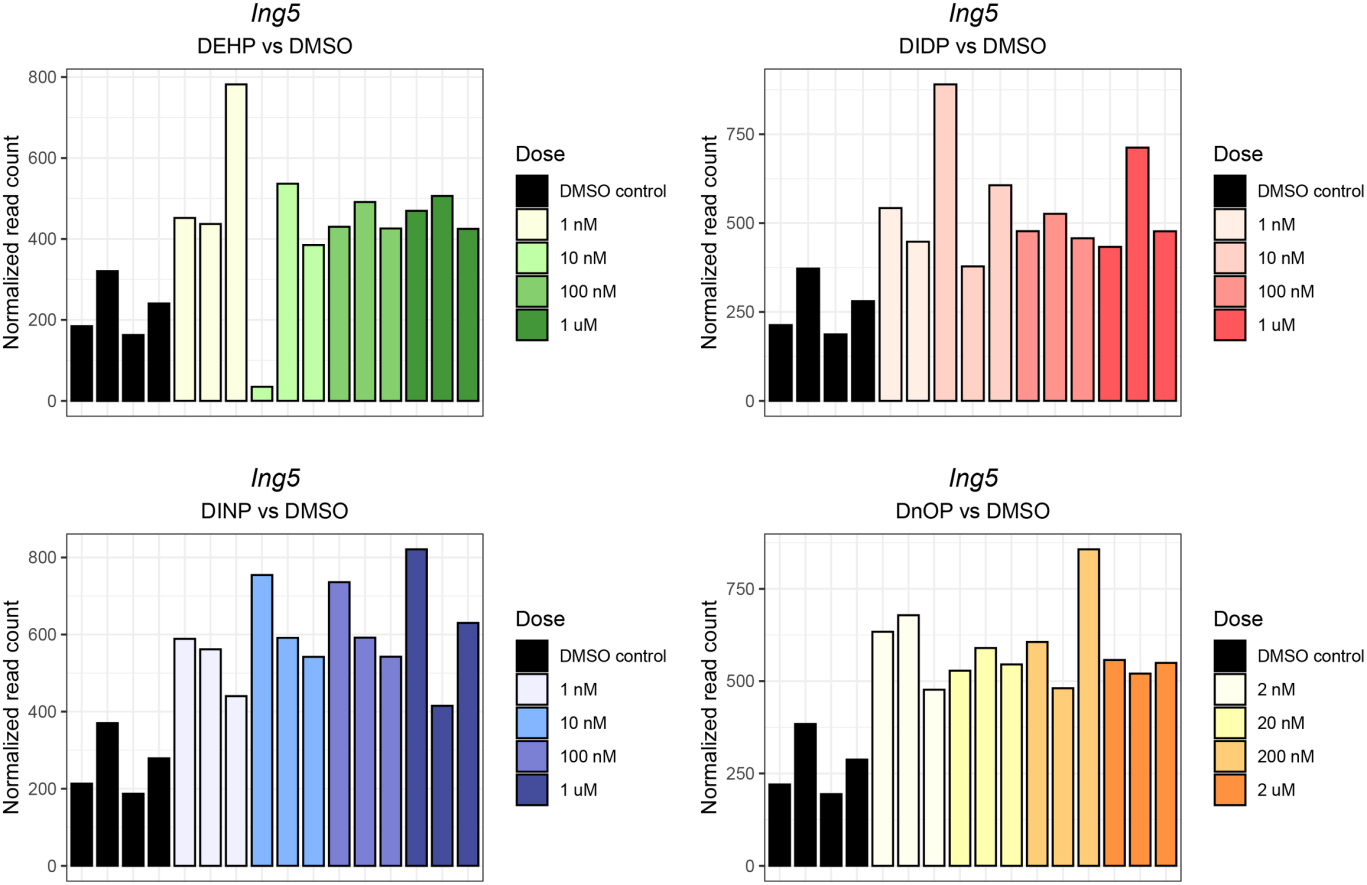
*Ing5* normalized expression in each phthalate and DMSO control sample. The different doses are reported in the legend. The darkest shade corresponds to the highest concentration (10 μM or 20 μM), while the lightest shades corresponds to the lowest (1 nM or 2 nM). The DMSO control samples are reported in black.

Interestingly, despite the weak effects on gene expression, the inhibitor of the *Ing5* gene was consistently upregulated in three out of four treatments (FDR: DEHP vs. DMSO = 4.44 * 10^−3^, DIDP vs. DMSO = 0.14, DINP vs. DMSO = 1.37 * 10^−3^, DnOP vs. DMSO = 1.23 * 10^−3^) (**Figure 3**).

Other genes differentially expressed in more than one condition were identified: *Acaa2* (DEHP, DINP, and DnOP vs. DMSO), *Plekha3* (DIDP and DnOP vs. DMSO), and five genes (*Cops2*, *Idh3g*, *Mid1*, *Rab5a*, *Zpf960*) dysregulated in DINP and DnOP vs. DMSO.

### 3.2 Gene set enrichment analysis

**Figure 4:**
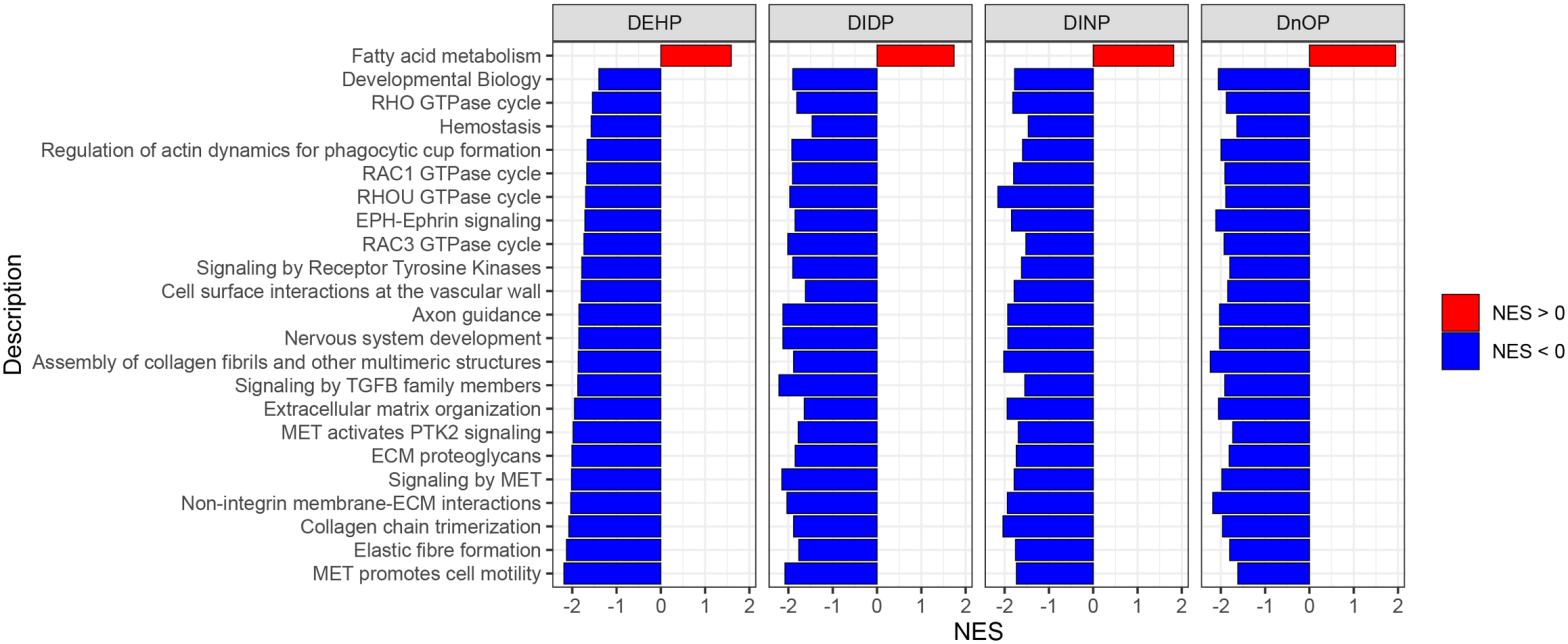
Results of gene set enrichment analysis (GSEA) on Reactome pathways. The reported pathways have a *q*-value of< 0.05 and appear in all four phthalates vs. DMSO comparisons.

GSEA was performed using the Reactome database with a *q*-value threshold of 0.05. We identified 123 enriched pathways in the DEHP vs. DMSO comparison, 79 in DIDP vs. DMSO, 173 in DINP vs. DMSO, and 311 in DnOP vs. DMSO (**Supplementary File 1**; **Supplementary Figure S5**). In both DEHP and DIDP vs. DMSO comparisons, most pathways were enriched in the control (normalized enrichment score NES< 0). Conversely, we observed a balance between pathways enriched in the treatment (NES > 0) and in the control in the DINP and DnOP vs. DMSO comparisons. To identify common effects across the treatments, we focused on the pathways that appeared in all comparisons (**Figure 4**), thus retrieving 23 terms, one enriched in the treatment and 22 enriched in the DMSO control.

Among the selected pathways with NES< 0, we identified several terms related to signal transduction and extracellular matrix (ECM) organization. The only term with NES > 0 is “fatty acid metabolism”. In **Supplementary Figure S6**, the terms are reported with their respective position in the Reactome term hierarchy for a better understanding of their relationships.

### 3.3 ATAC-Seq results

As explained in the previous section, *Ing5* upregulation was observed in three phthalate exposures out of four. ING5 is a component of the histone acetyltransferase complexes HBO1-JADE, which mediates histone H4 acetylation *in vivo*, and HBO1-BRPF1 and MOZ/MORF, which mediate histone H3 acetylation (43, 44). For this reason, we investigated whether phthalate treatment could have an impact on the chromatin status with ATAC-Seq. To this end, we selected two of the four phthalates, DEHP and DINP, and the highest dose included in the differential expression analysis (1 μM). The exposure was increased to 5 days to allow time for any chromatin rearrangements, if any, to take place, accounting for any delay between gene upregulation of *Ing5* and an actual observable effect on the epigenome. For ATAC-Seq library preparation, a viability of at least 90% was required. Unfortunately, we were not able to recover enough cells from our thyroid follicles model with this viability. For this reason, we selected the human epithelial thyroid cell line Nthy-ori 3-1 (**Supplementary Figure S7**). In the next sections, some quality control metrics of the ATAC-Seq libraries and the results of the differential accessibility analysis are reported.

### 3.4 ATAC-Seq QC

ATAC-Seq libraries had a median of 82.2 M reads per sample (min = 26.5, max = 237.9 M) (**Supplementary Figure S8A**), with a median of 77.36% (min = 75.46%, max = 79.08%) of sequenced reads being successfully aligned to the GRCh38 nuclear genome (**Supplementary Figure S8B**). The transcription start site (TSS) enrichment score had a distribution between 10.7 and 19.5 (median: 18.1) (**Supplementary Figure S8C**). The distribution of nucleosome-free regions (NFR), mono-, di-, tri-, or poly-nucleosome regions, was consistent across samples (**Supplementary Figure S8D**), as were the read length distribution profiles typical of ATAC-Seq libraries (**Supplementary Figure S8E**). The library complexity metrics were within the accepted values recommended by the Encode Project (**Supplementary Figure S8F**) (76).

### 3.5 Differential accessibility analysis by ATAC-Seq

**Figure 5:**
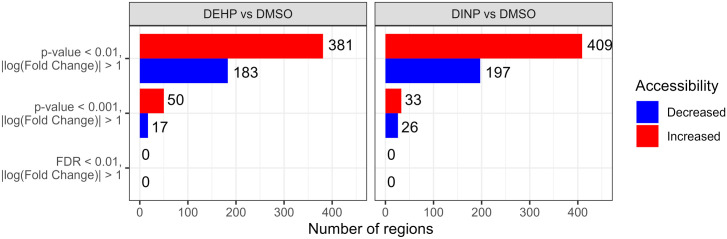
Number of differentially accessible regions with *p*-value of< 0.001 or 0.01 and |log(fold change)|>1 in the DEHP- or DINP-treated samples vs. DMSO control. After multiple-testing correction, none of the regions had an FDR of< 0.01.

We identified 111,133 genomic regions when comparing DEHP-treated and DMSO samples, and 118,855 regions in the DINP vs. DMSO comparison were tested for differential accessibility. In both treatments, we observed a general increase in accessibility compared to the control, but none of the regions passed multiple-testing correction (**Figure 5**).

We annotated the regions with a *p*-value of< 0.01 with HOMER to identify the closest gene to each region and looked for overlap between DEHP vs. DMSO and DINP vs. DMSO. We identified four regions with decreased accessibility and 17 with increased accessibility that overlap with regulatory regions (**Supplementary Table S3**).

We looked at which of these regions fall within the TSS or transcription termination site (TTS). We identified four regions, all with increased accessibility: two located at the TSS of the cell division cycle associated 2 (*CDCA2*) and complexin 1 (*CPLX1*) genes, and two located at the TTS of cyclin I (*CCNI*) and dystrobrevin-binding protein 1 (*DTNBV1*). However, when inspecting the normalized read coverage on the IGV, there did not seem to be a clear increase in accessibility compared to the control (**Supplementary Table S4**; **Supplementary File 2**).

## 4 Discussion

In this work, we analyzed the response of mouse embryonic stem cell-derived thyroid follicles after exposure to the phthalates DEHP, DIDP, DINP, and DnOP in a range of concentrations from 1 nM to 10 μM (2 nM to 20 μM for DnOP) for 24 h. The low dose range was selected to reflect the low daily intake of phthalates measured in the general population (77) and the relatively short exposure time to detect the initial reaction to phthalate exposure by identifying the early changes in the transcriptome. In this way, we aimed to test whether our model would be able to capture the molecular initiating event (MIE) of these phthalates, which would then be followed by the key events (KEs) to ultimately lead to an adverse outcome (integrated in the concept of an adverse outcome pathway (AOP)) (78). We performed RNA-Seq analysis and simultaneously analyzed both mRNA and small RNAs from the same samples. The dose series analysis showed how most of the identified genes either increased or decreased sharply in expression at the highest dose, setting it apart from the others and possibly indicating it as a dose to determine a PoD for those genes, which is used in toxicology to establish a threshold dose for risk assessment (75, 79).

The compounds showed a modest effect on the cells at the time and doses of exposure in terms of the number of differentially expressed genes and miRNAs, while no effect was observed on snoRNA expression. DIDP was the only compound where a microRNA (mmu-miR-143-3p) was downregulated. This microRNA, together with mmu-miR-143-5p, has been observed to be downregulated in several cancers and is thought to have tumor-suppressing activity and be a negative regulator of cell proliferation (80–82). Despite the low number of DEGs, we observed a partial overlap across treatments (*Acaa2* and *Plekha3* in three treatments, *Cops2*, *Idh3g*, *Mid1*, *Rab5a*, and *Zpf960* in two treatments). It is possible that the higher number of DEGs in the DnOP vs. DMSO comparison could be explained by the doses used being twice as high as the other phthalates, though still within the same order of magnitude. Acetyl-CoA acyltransferase 2 (Acaa2) is one of the enzymes that catalyzes the last step of the mitochondrial beta-oxidation pathway. Pleckstrin homology domain containing A3 (Plekha3) is involved in the regulation of vesicular cargo transport from the trans-Golgi network to the plasma membrane and is predicted to be involved in ceramide transport and intermembrane lipid transfer (83, 84). Cops2 is a member of the COP9 signalosome complex (CSN), which is involved in decreasing the ubiquitin ligase activity of the SCF-type E3 ligase complexes. Idh3g is an enzyme that takes part in the Krebs cycle and performs the decarboxylation of isocitrate into alpha-ketoglutarate. Midline 1 (Mid1) is likely involved in the formation of multiprotein structures acting as anchor points to microtubules. It has also E3 ubiquitin ligase activity toward the protein Igbp1, promoting its degradation. Rab5a is a member of the RAS oncogene family and is a small GTPase that, in its active form, recruits proteins responsible for vesicle formation, movement, tethering, and fusion (83).

Via GSEA, we looked for enriched pathways shared by the four treatments to try and identify effects that could be attributed to the “phthalate” EDC class. Only the pathway fatty acid metabolism was enriched in all treatments. Interestingly, phthalates have been shown to increase the metabolism of fatty acids not only in the liver (85, 86) but also in nonliver tissue such as cardiomyocytes, where increased use of fatty acids for energy production was suggested (87). To our knowledge, our analysis is the first observation of the conservation of these mechanisms in an *in vitro* thyroid model. Additionally, among the pathways downregulated in the treatment groups, we found many related to cell–ECM organization and receptor signaling. It is also interesting to note that despite the low number of DE genes due to the low doses used, were still able to detect relevant enriched pathways using GSEA.

Taken together, the results of differential gene expression gene analysis and GSEA seem to point to an effect of phthalates on energy production, with genes involved in cellular respiration being dysregulated and lipid metabolism increasing.

*Ing5* was upregulated in three treatments (DEHP, DINP, DnOP) compared to the control. The ING family comprises five genes (*ING1* to *ING5*), which have a role in cell cycle regulation and cell proliferation by interacting with several partners, such as p53, p300, and histone acetylation complexes (43, 88). *ING5* is a tumor-suppressor gene that is downregulated in several types of cancer, including thyroid (89), colorectal (90), breast (91), and lung (92). Its protein is a component of the histone acetyltransferase HBO1-JADE, which acetylates histone H4 at Lys residues 5, 8, and 12 (H4K5ac, H4K8ac, H4K12ac), MOZ/MORPH, which performs histone H3 acetylation, and HBO1-BRPF (H3K14ac) (44, 93).

As a consequence of *Ing5* overexpression, we hypothesized that phthalate treatment could have an impact on chromatin status. For this reason, we exposed the human thyroid follicular epithelial cell line Nthy-ori 3-1 to 1 μM of DEHP or DINP for 5 days and analyzed the genomic accessibility by ATAC-Seq. We reasoned that, since *Ing5* is not a thyroid-specific gene and its expression is not limited to the thyroid, we would be able to observe changes in a different cell model since we would be investigating a general phthalate mechanism rather than a model-specific response. Differential accessibility analysis resulted in a general increase in accessibility in the treatment group, but none of the identified regions passed multiple-testing correction. Among the regions with a *p*-value of< 0.01, we identified four common ones with increased accessibility in the DEHP vs. DMSO and DINP vs. DMSO comparison localized on regulatory regions in the TSS or TTS. However, the signal did not seem to reflect a real change in accessibility.

In this work, we showed that even with a stem cell-derived *in vitro* thyroid model exposed to a range of low, biologically relevant concentrations of four phthalates, we were able to detect some of the effects that have been previously reported *in vivo*. Our analysis demonstrates that it is not necessary to use cytotoxic doses in toxicological experiments to obtain observable results and that low-dose exposure can be analyzed without lowering the statistical stringency. We are convinced that 3D *in vitro* systems, such as organoids, can be a valid alternative to animal studies even for EDCs, provided that enough datasets are generated to allow regulators to infer risk assessment thresholds.

## Data availability statement

The datasets presented in this study can be found in online repositories. The names of the repository/repositories and accession number(s) can be found below: https://www.ebi.ac.uk/arrayexpress/, E-MTAB-12830.

## Author contributions

Conceptualization: MN and FC. Methodology: MN, MR, DH, and DC. Formal analysis and investigation: MN. Writing—original draft preparation: MN and FC. Writing—review and editing: all coauthors. Funding acquisition: FC, SC, SG, and LM. Resources: FC, SC, SG, and LM. Supervision: FC.
