# Supplementary material for: Investigation of the effects of phthalates on in vitro thyroid models with RNA-Seq and ATAC-Seq
Source: Front Endocrinol (Lausanne). 2023 Sep 22;14:1200211. doi: 10.3389/fendo.2023.1200211 (PMC10556862; doi:10.3389/fendo.2023.1200211)
Supplement: Supplementary file 1 [file DataSheet_1.pdf]

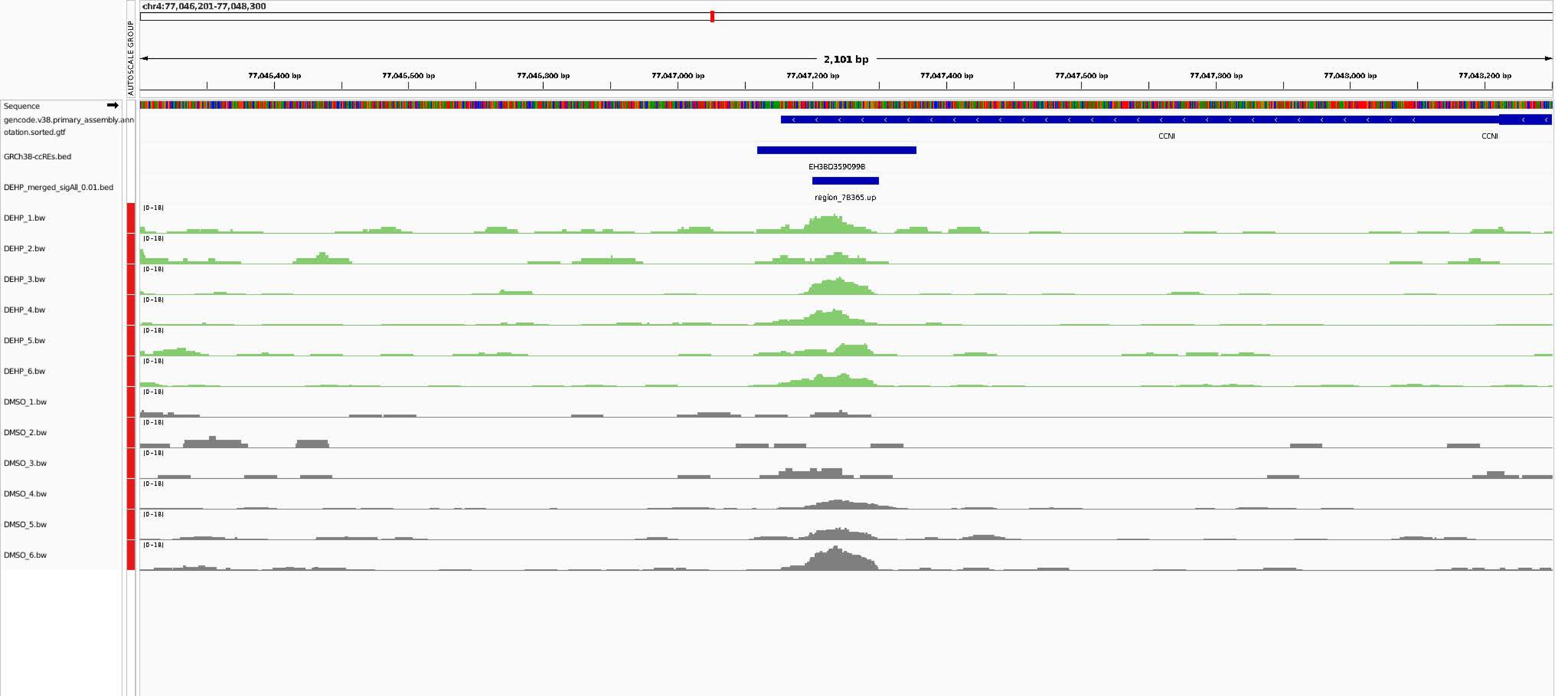

IGV screenshot of the four regions identified in Supplementary Table 4. The tracks have been normalized for visualization with BeCorrect

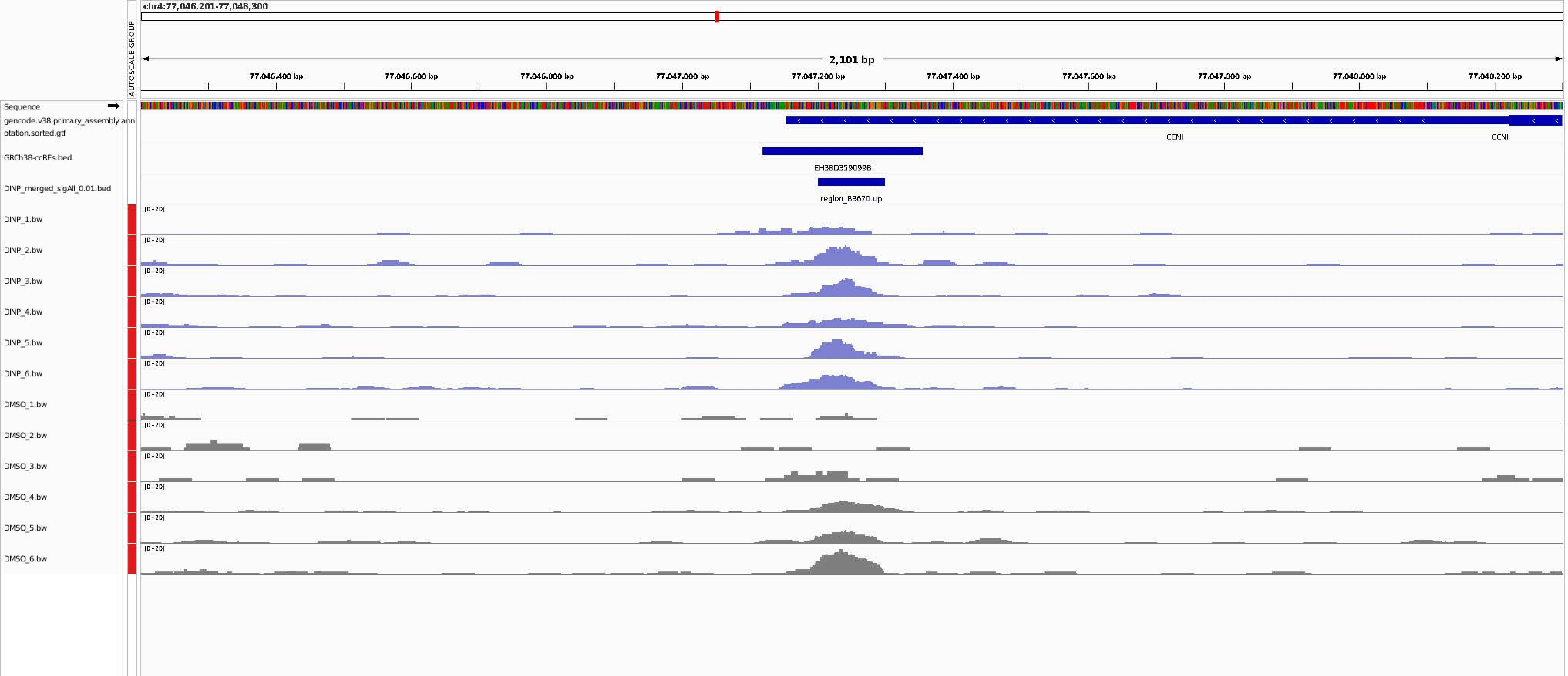

IGV screenshot of the four regions identified in Supplementary Table 4. The tracks have been normalized for visualization with BeCorrect

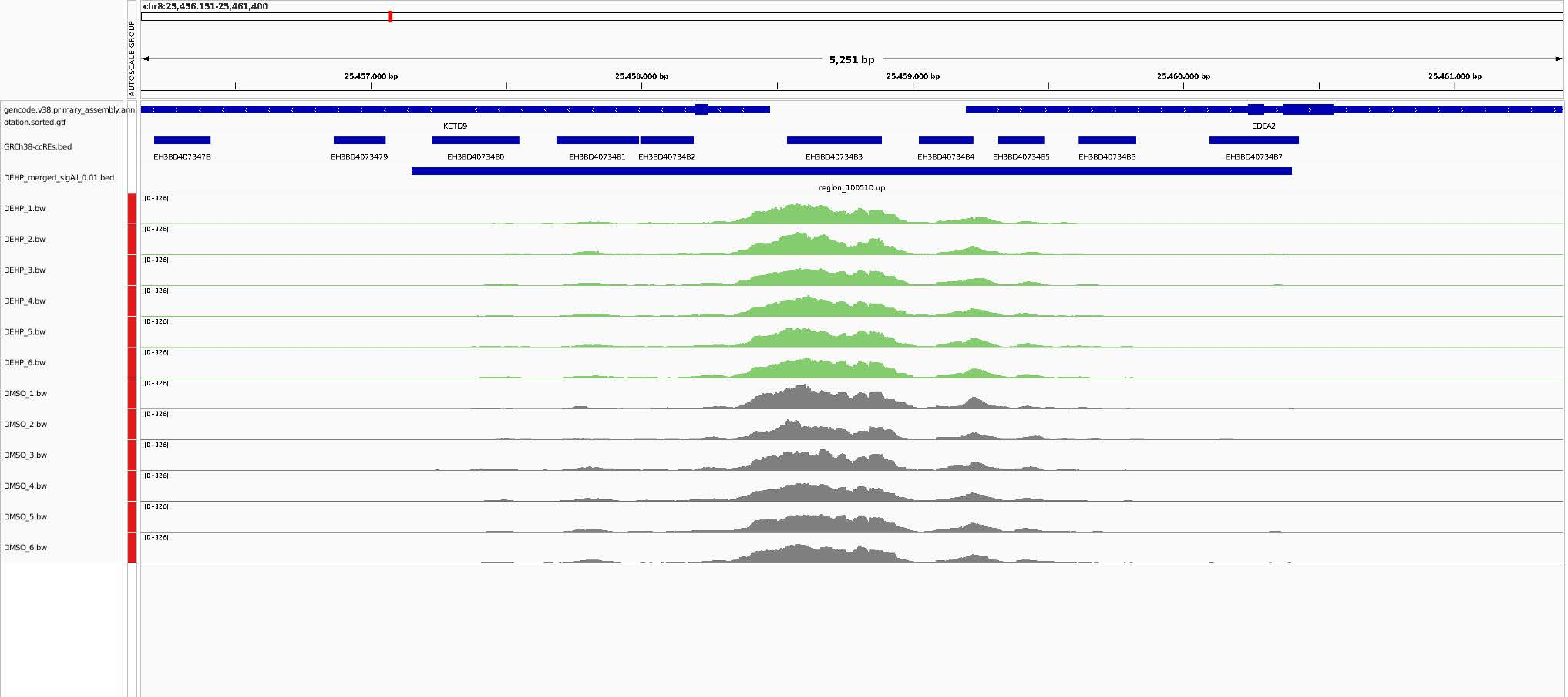

IGV screenshot of the four regions identified in Supplementary Table 4. The tracks have been normalized for visualization with BeCorrect

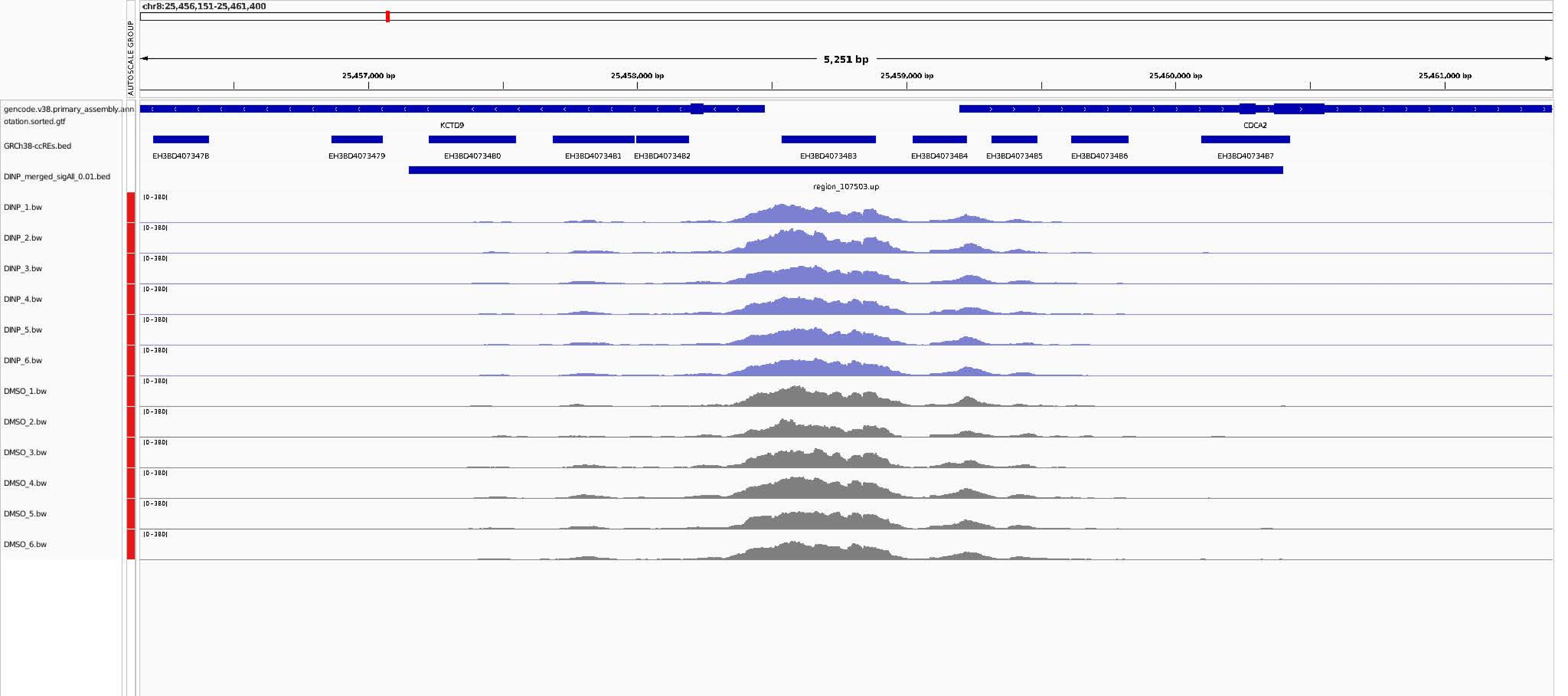

IGV screenshot of the four regions identified in Supplementary Table 4. The tracks have been normalized for visualization with BeCorrect

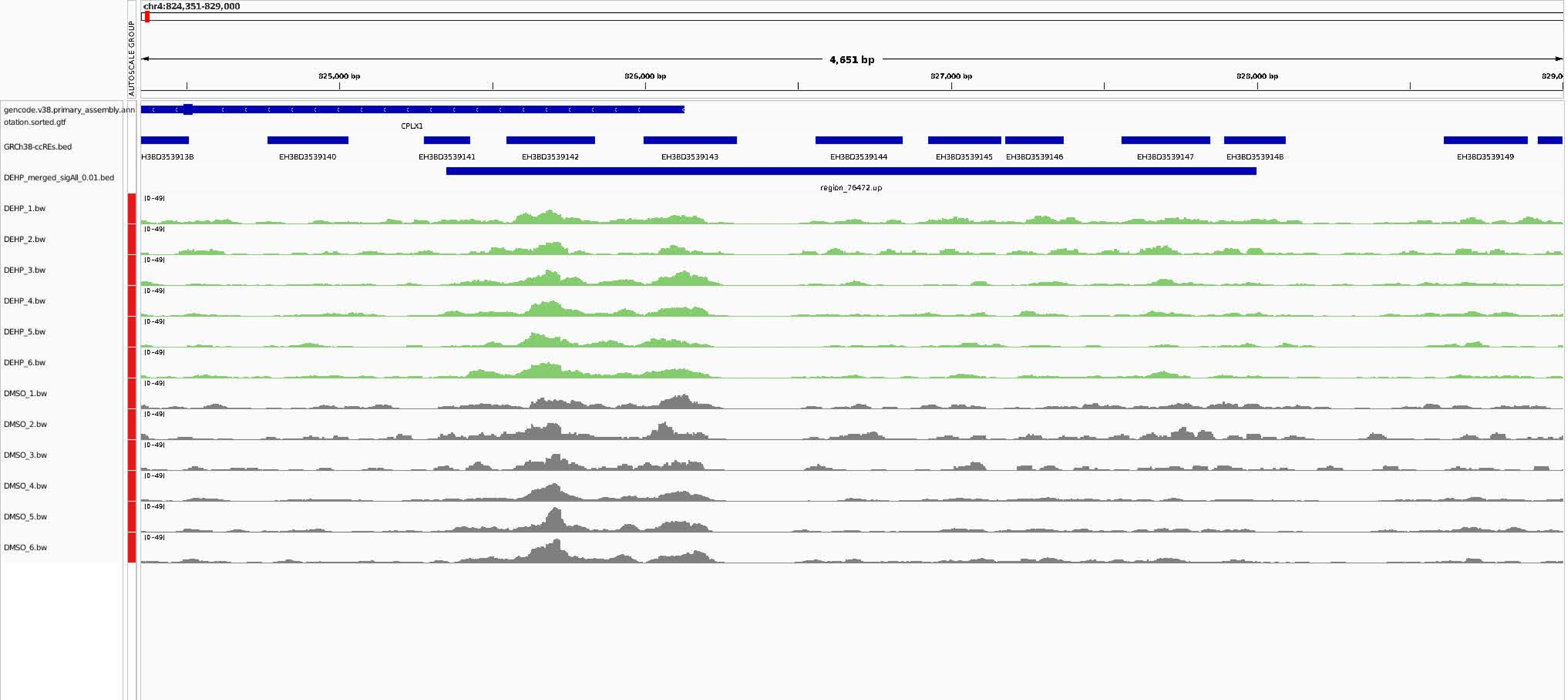

IGV screenshot of the four regions identified in Supplementary Table 4. The tracks have been normalized for visualization with BeCorrect

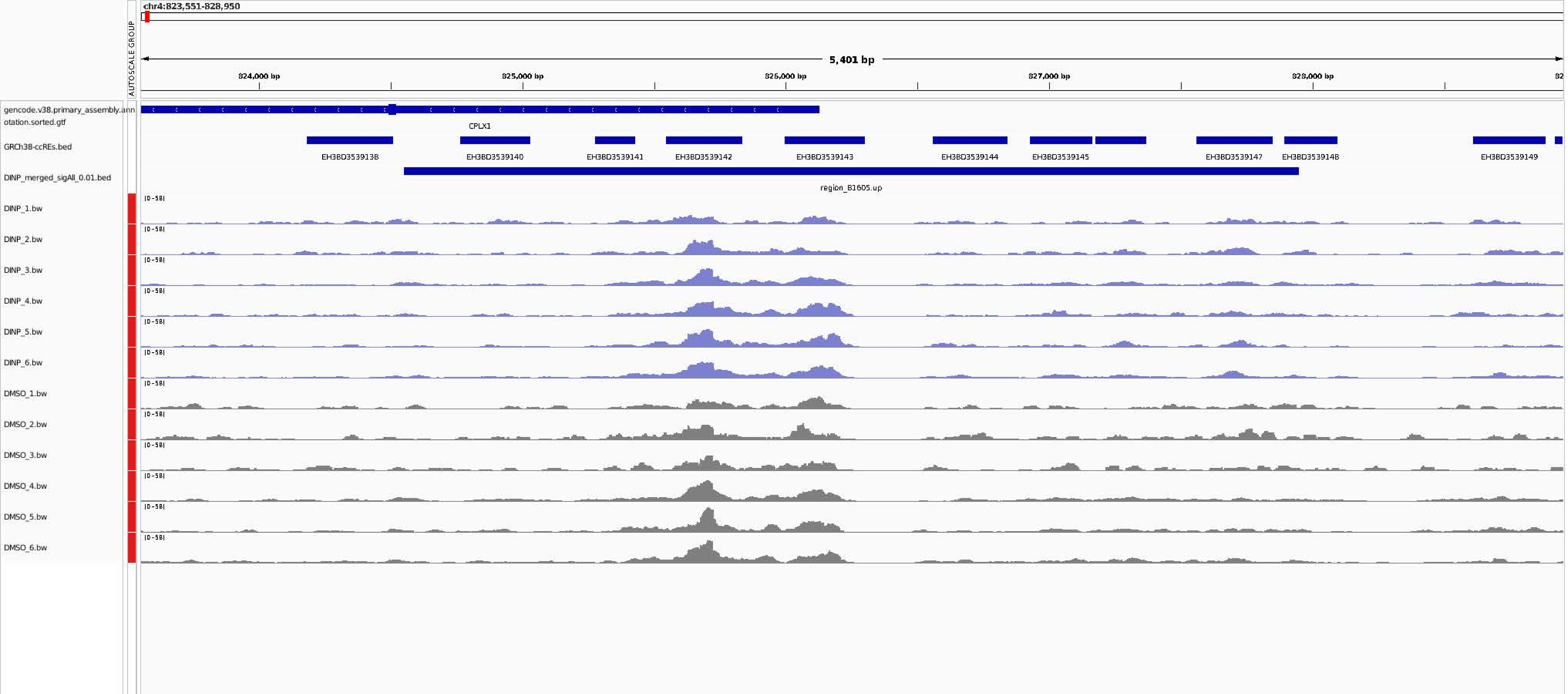

IGV screenshot of the four regions identified in Supplementary Table 4. The tracks have been normalized for visualization with BeCorrect

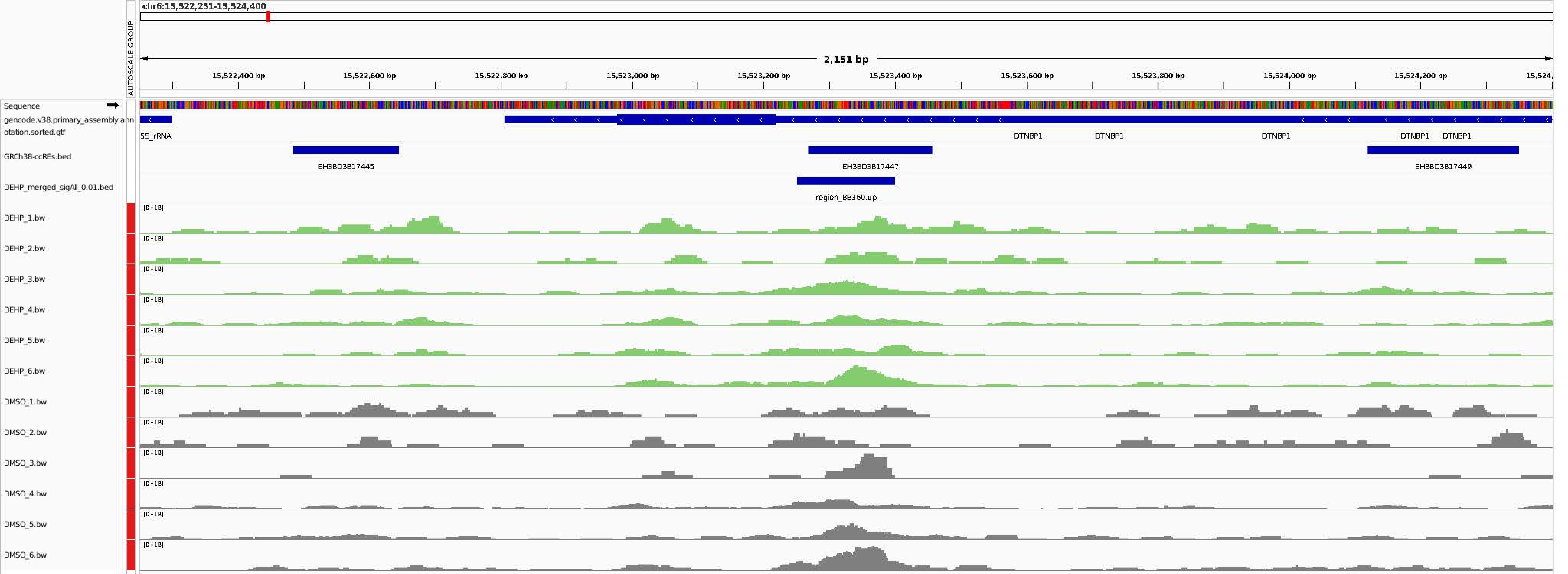

IGV screenshot of the four regions identified in Supplementary Table 4. The tracks have been normalized for visualization with BeCorrect

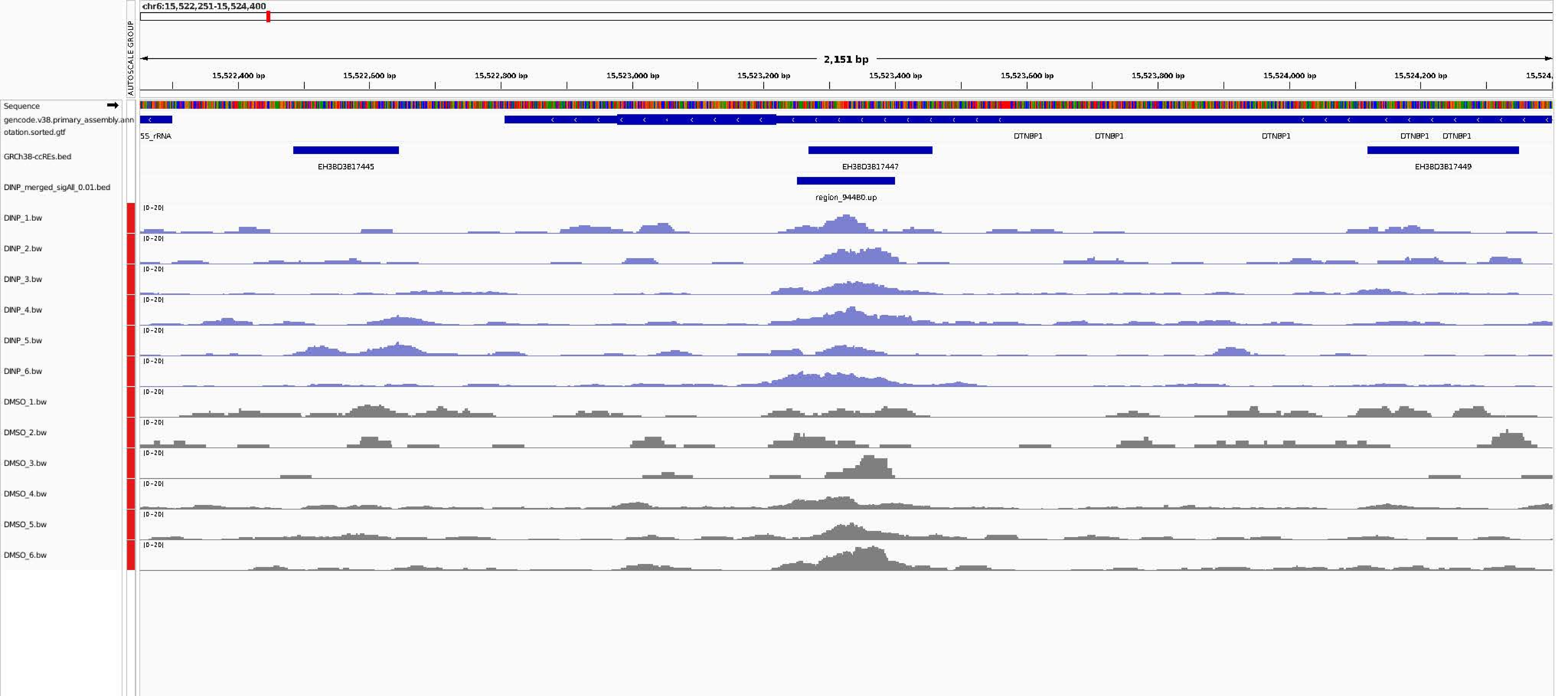

IGV screenshot of the four regions identified in Supplementary Table 4. The tracks have been normalized for visualization with BeCorrect
