# Supplementary material for: Investigation of the effects of phthalates on in vitro thyroid models with RNA-Seq and ATAC-Seq
Source: Front Endocrinol (Lausanne). 2023 Sep 22;14:1200211. doi: 10.3389/fendo.2023.1200211 (PMC10556862; doi:10.3389/fendo.2023.1200211)
Supplement: Supplementary file 3 [file DataSheet_3.docx]

Supplementary Material

Investigation of the effects of phthalates on in vitro thyroid models with RNA-Seq and ATAC-Seq

Marta Nazzari^1^, Mírian Romitti^2^, Duncan Hauser^1^, Daniel J. Carvalho^3^, Stefan Giselbrecht^3^, Lorenzo Moroni^3^, Sabine Costagliola^2^, Florian Caiment^1,*^

*** Correspondence:** Corresponding Author: florian.caiment@maastrichtuniversity.nl

# Supplementary Methods

## Embryonic stem cells culture and differentiation

Thyroid follicles were differentiated from the A2Lox.Cre_TRE-Nkx2-1/Pax8_Tg-EGFP mouse ESC as previously described [42,47]. Briefly, cells were initially cultured on gamma-irradiated mouse embryonic fibroblasts (MEFs) feeder using mouse stem cell medium [42,47] and incubated at 37 °C, 5% pCO2 and >95% humidity. For differentiation into thyroid, embryoid bodies (EBs) were generated by hanging drops culture of ESCs (1000 cells per drop) for 4 days. They were then collected and embedded in growth factor restricted Matrigel (354230, Corning). 50 mL matrigel drops containing around 20 EBs were plated into 12-well plates. Cells were differentiated using a differentiation medium composed of DMEM (31966021, Gibco) supplemented with 15% FBS, vitamin C (50 μg mL-1) (A4544, Sigma), nonessential amino acids (0.1 mM) (11140035, Gibco), sodium pyruvate (1 mM) (11360039, Gibco), penicillin and streptomycin (50 U mL-1) (15140122, Gibco), 2-mercaptoethanol (0.1 mM) (31350010, Gibco). The differentiation medium was supplemented with 1 μg mL-1 of doxycycline (D9891-1G, Sigma) for 3 days for Nkx2-1 and Pax8 induction, followed by two weeks of maturation by using the differentiation medium containing 8-Br-cAMP (0.3 nM) (B 007-500, BioLog).

## Follicles enrichment protocol

At day 21, after complete thyroid maturation, matrigel drops containing the thyroid follicles were washed twice with Hanks’s balanced salt solution (HBSS, containing calcium and magnesium) (14025050, Gibco) and incubated in a HBSS solution (1 mL per well) containing 10 U mL-1 of dispase® II (4942078001, Roche) and 125 U mL-1 of collagenase type IV (Sigma) for 30-45 min at 37 °C. The enzymes were then inactivated by adding 10% FBS. Organoids were centrifuged at 1200 rpm for 3 minutes. After rinsing twice with HBSS, the follicles were enriched by filtration using 30 mm (to remove single cells) (43-50030, pluriSelect Life Science GmbH) to and 100 mm reverse strainer (to remove big follicles aggregates) (43-50100, pluriSelect Life Science GmbH).

Resuspended follicles were cultured in subsequent experiments in the differentiation medium described above and supplemented with 8-Br-cAMP (10 µM) and TGF-βRI inhibitor SB431542 (10 µM) (1614, Tocris), hereafter termed “supplemented differentiation medium”.

# Supplementary Figures and Tables

## Supplementary Figures


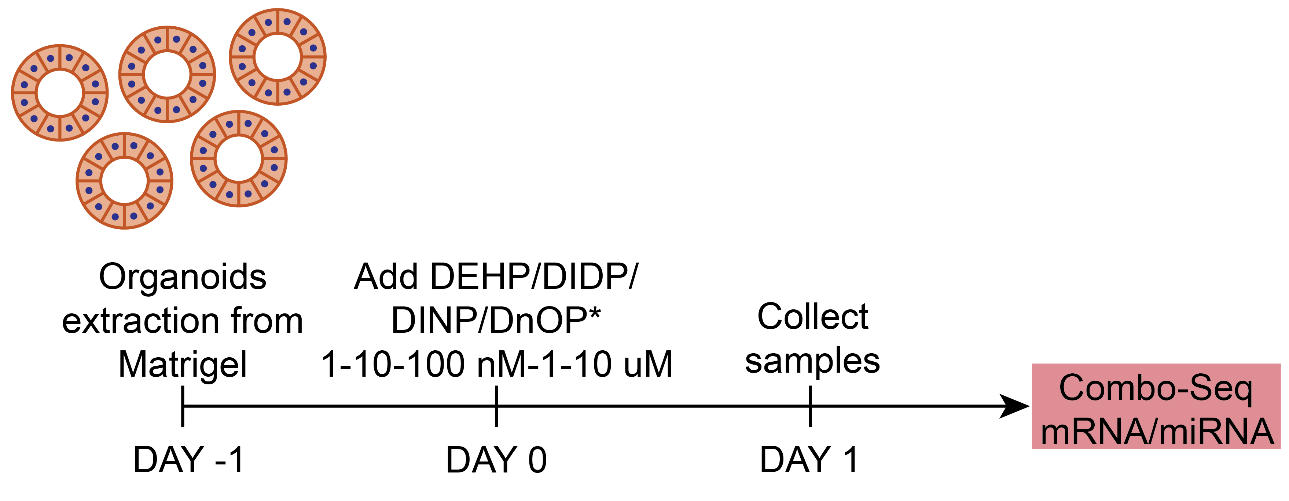


**Supplementary Figure 1.** Graphical representations of the exposure regimens of thyroid follicles to the phthalates (* = for DnOP the concentrations used were 2-20-200 nM-2-20 μM).


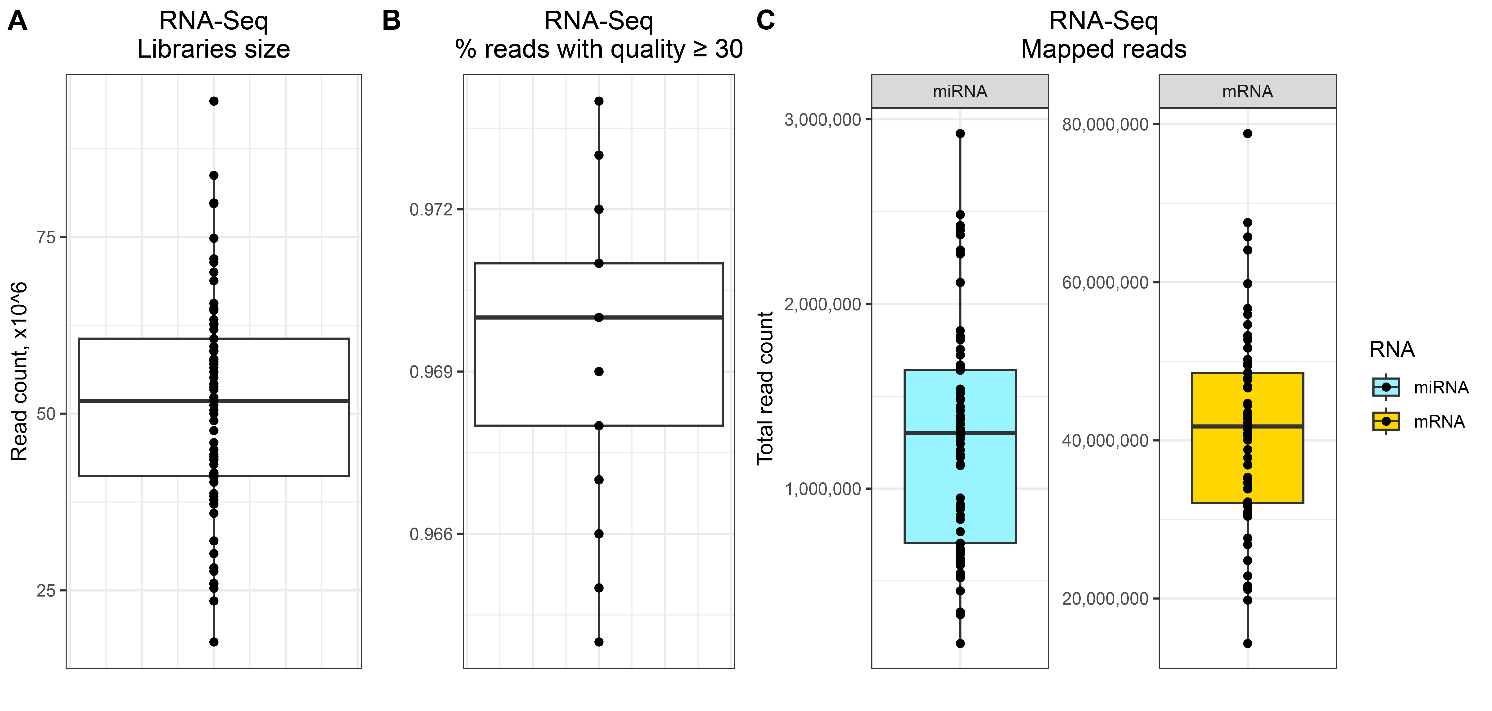


**Supplementary Figure 2.** RNA-Seq dataset metrics. (A) Number of sequenced reads. (B) Percentage of sequenced reads with quality score Q30>=30. (C) Number of reads mapped to miRNAs (light blue) or mRNAs (yellow). Each dot represents a sample.


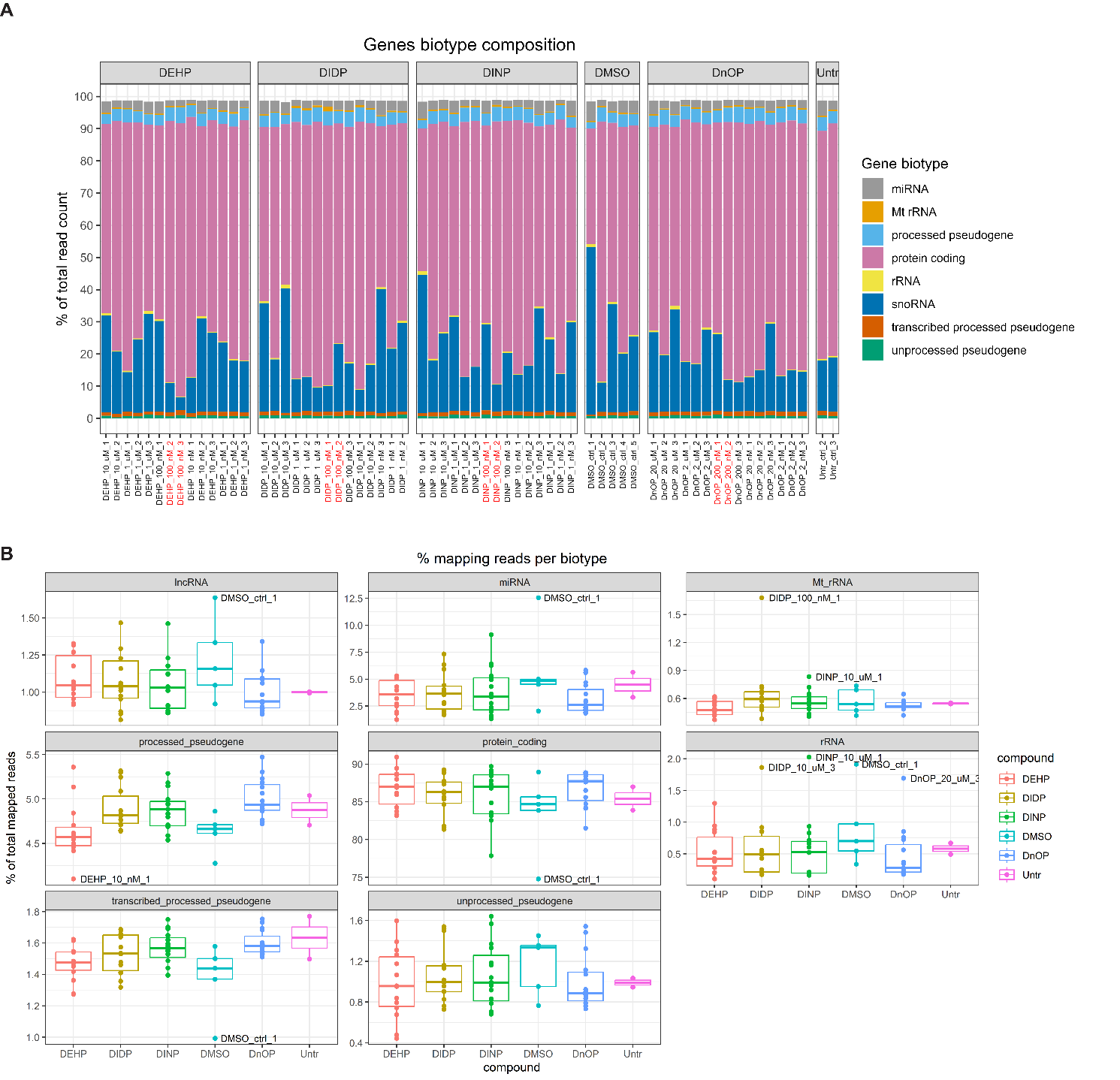


**Supplementary Figure 3.** (A) Genes biotype composition of RNA-Seq samples expressed as percentage of total normalized read count. For clarity and to reduce noise, only the biotypes that make up at least 1% of the total in at least one sample are reported. The sample names for which 14 cycles during the library prep protocol were used are reported in red. (B) Biotype distribution after snoRNA removal. The biotypes that make up at least 1% of the mapped reads are reported. The labelled samples are the ones flagged as outliers.

**
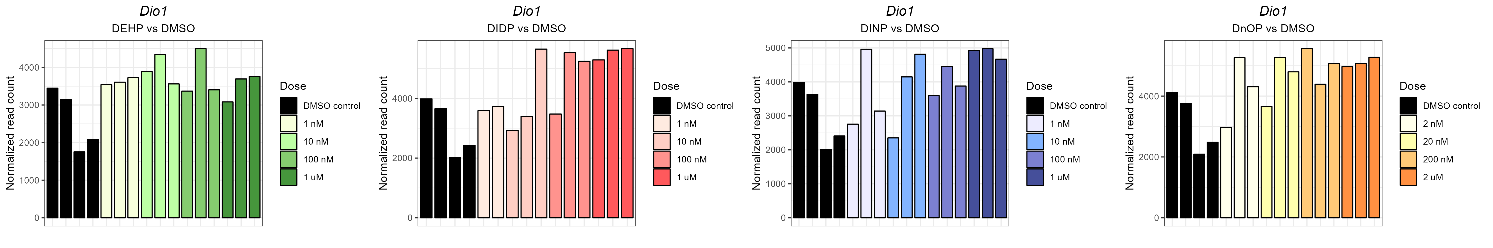

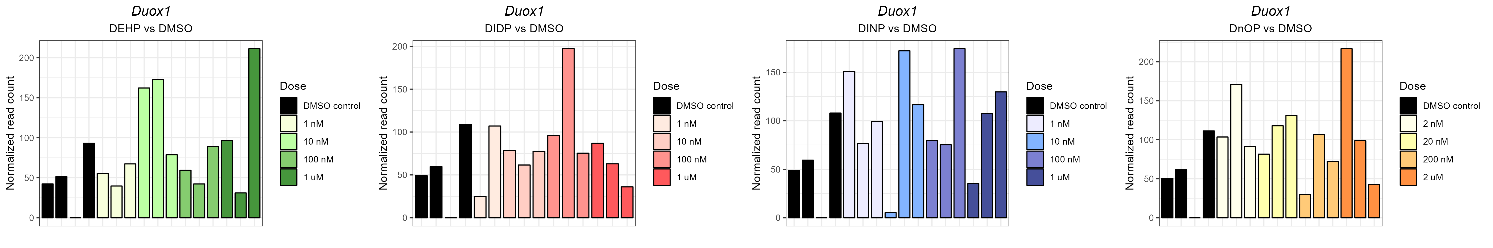

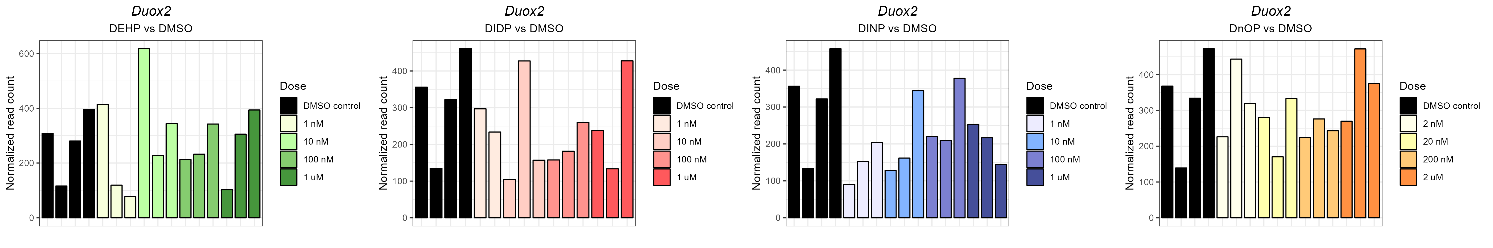

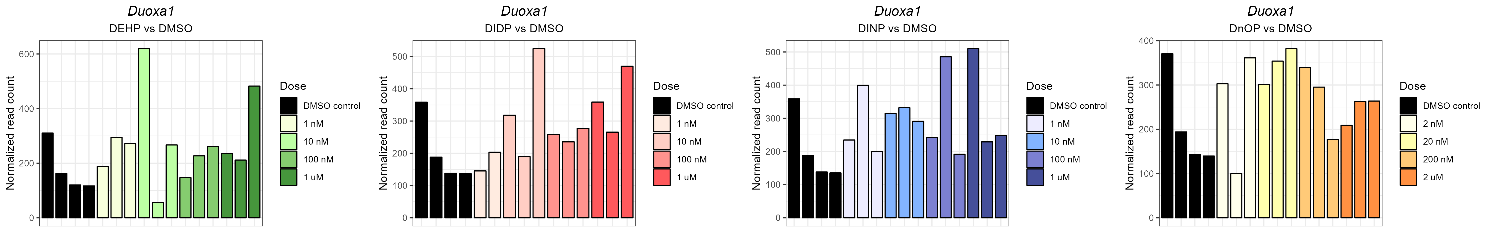

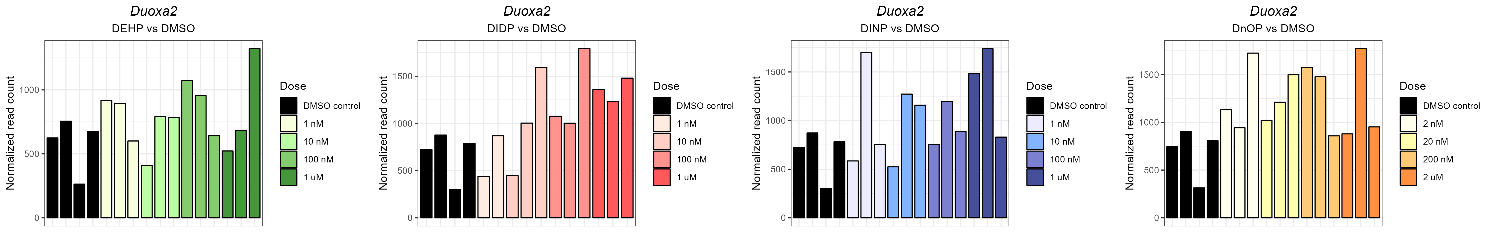

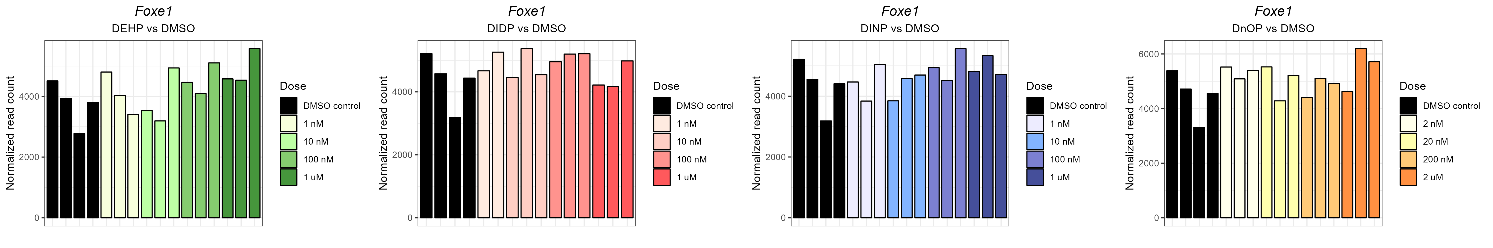

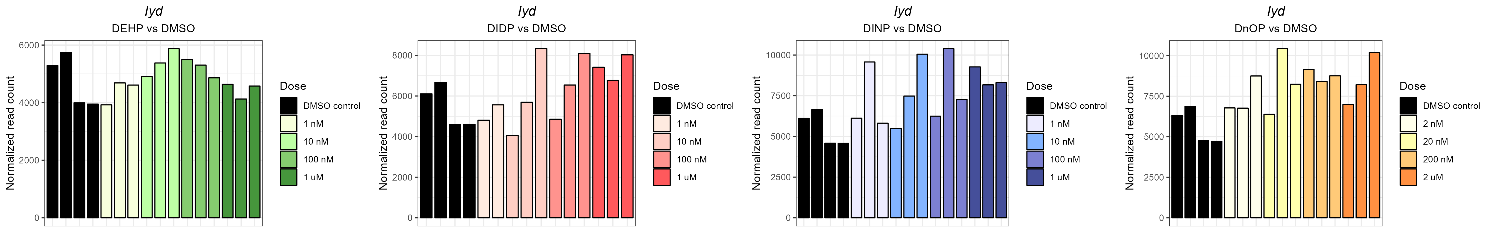

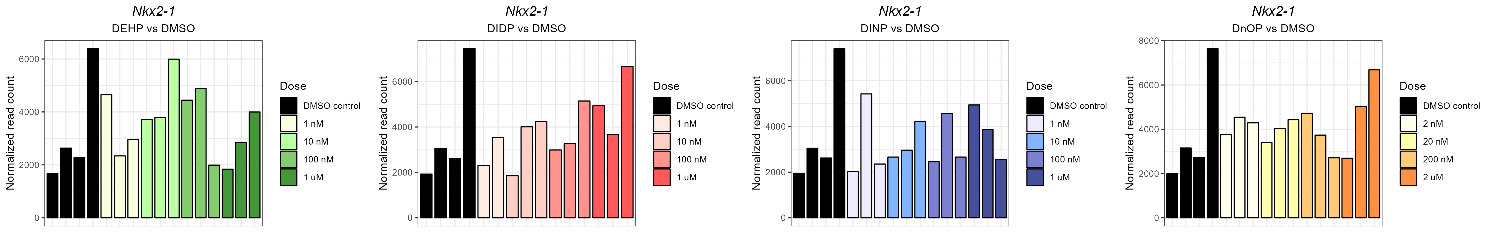

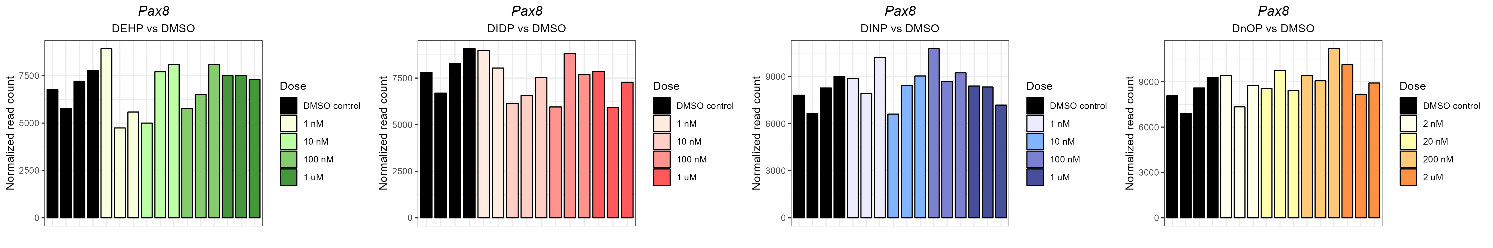

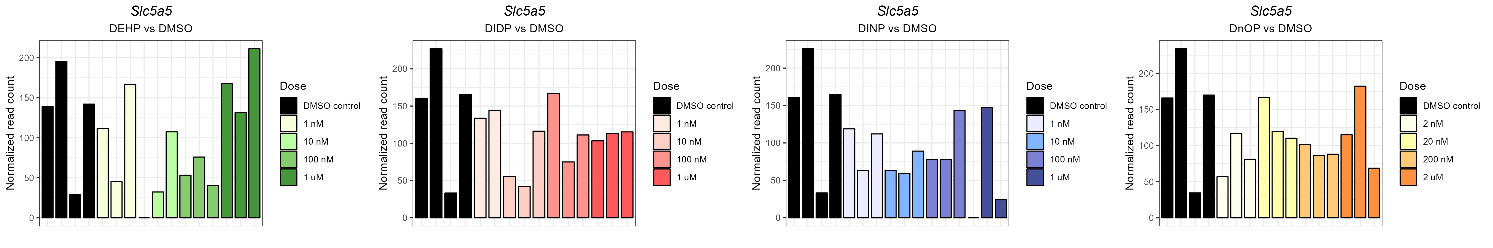

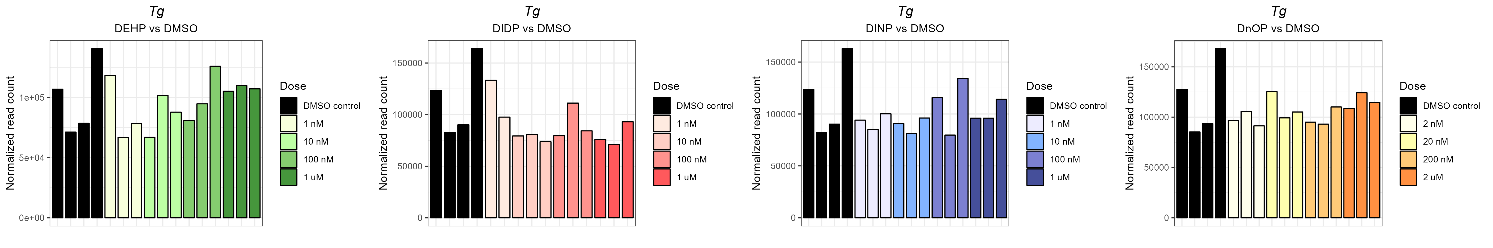

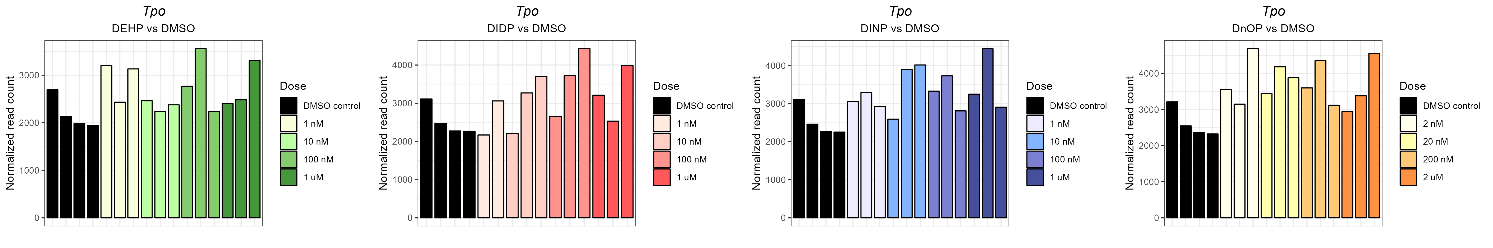

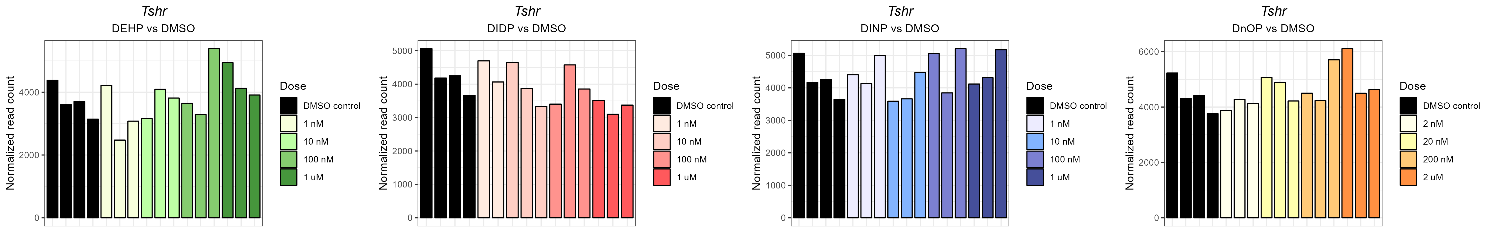
** **Supplementary Figure 4**. Barplots of normalized expression of some thyroid markers in each phthalate and DMSO control samples. the gene name is indicated on top of each plot and the different doses are reported in the legend. The darkest shade corresponds to the highest concentration (1 or 2 μM), while the lightest shades to the lowest (1 or 2 nM). The DMSO control samples are in black.


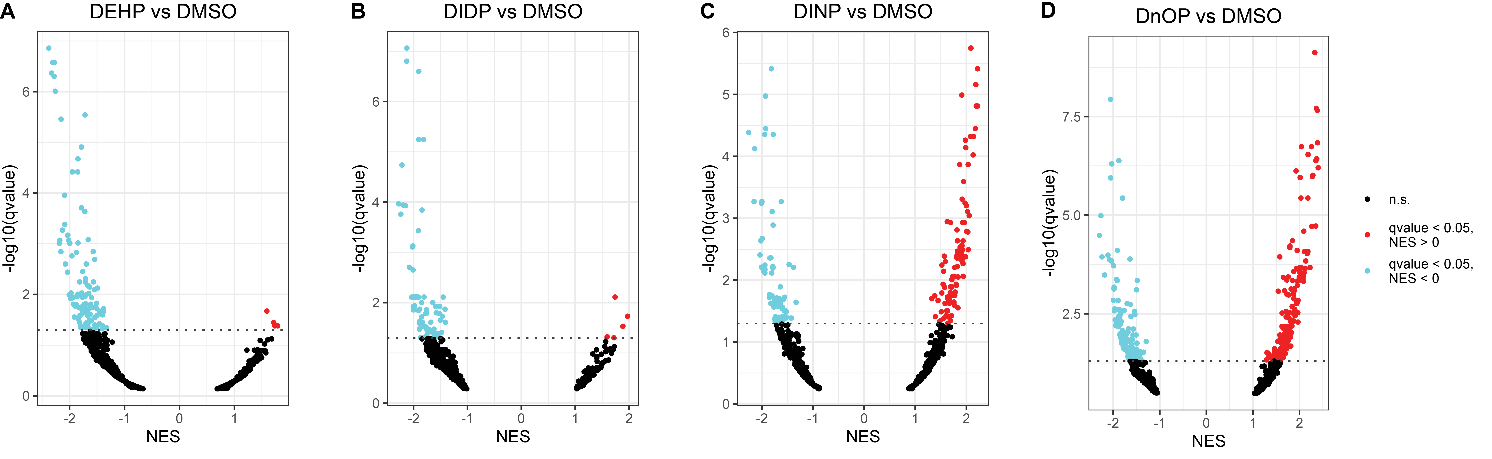


**Supplementary Figure 5.** Volcano plots of the enriched Reactome pathways identified by GSEA analysis in each phthalate vs DMSO control (A: DEHP vs DMSO; B: DIDP vs DMSO; C: DINP vs DMSO; D: DnOP vs DMSO). Every dot represents a pathway. Pathways with a q-value<0.05 and normalized enrichment score (NES)>0 are in red. Pathways with a q-value<0.05 and NES<0 are in cyan. Pathways with q-value>=0.05 are in black (‘n.s.’). The q-value threshold of 0.05 is indicated as a dotted line on the y-axis.


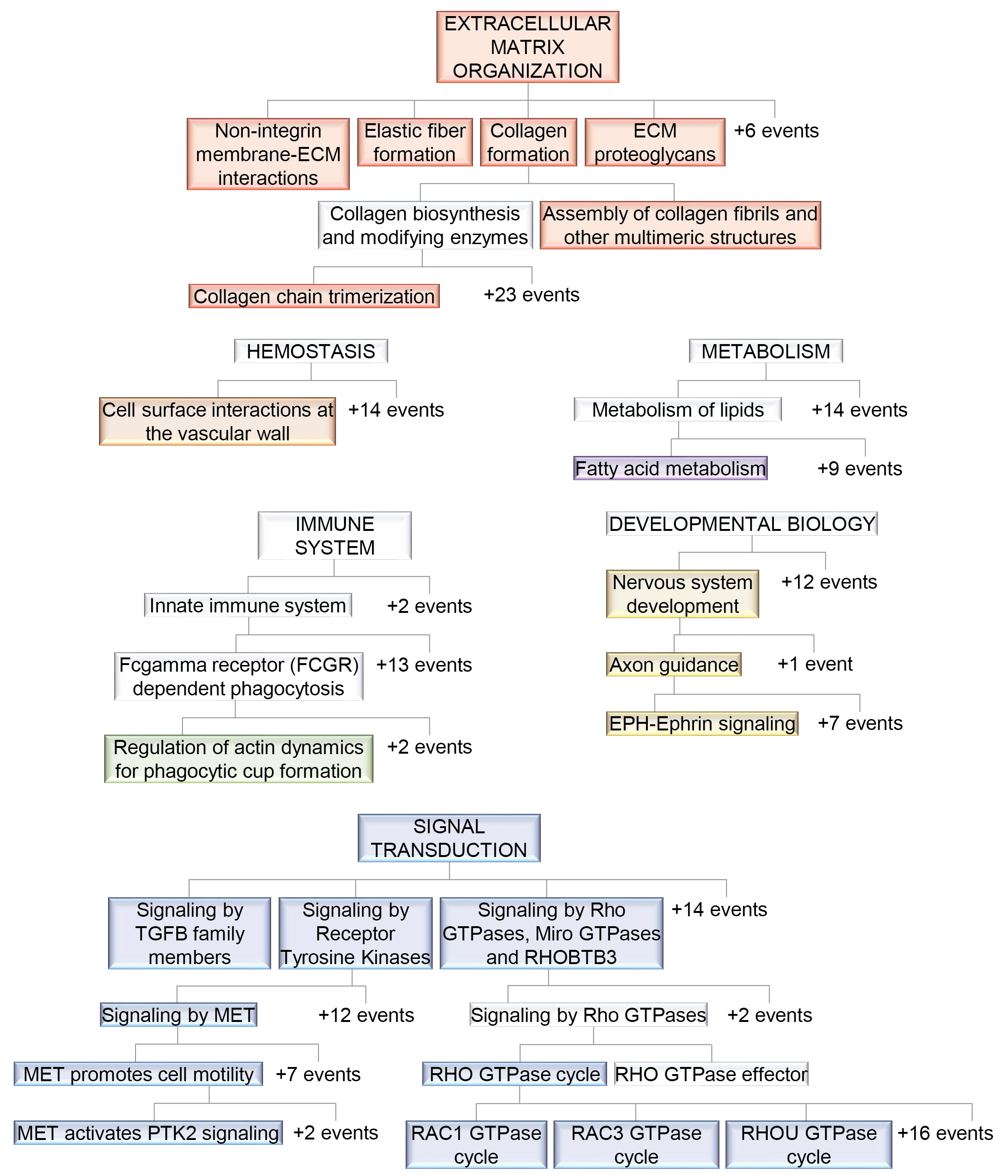


**Supplementary Figure 6.** Common enriched Reactome pathways in all four phthalates identified by GSEA. The top term, in uppercase, represents the most parent term in the hierarchy. The colored terms are the ones reported in Figure 4, while the terms in a white rectangle are reported for better understanding the relationships among terms. If a branch has more children events, it is indicated as “+N events”.

**
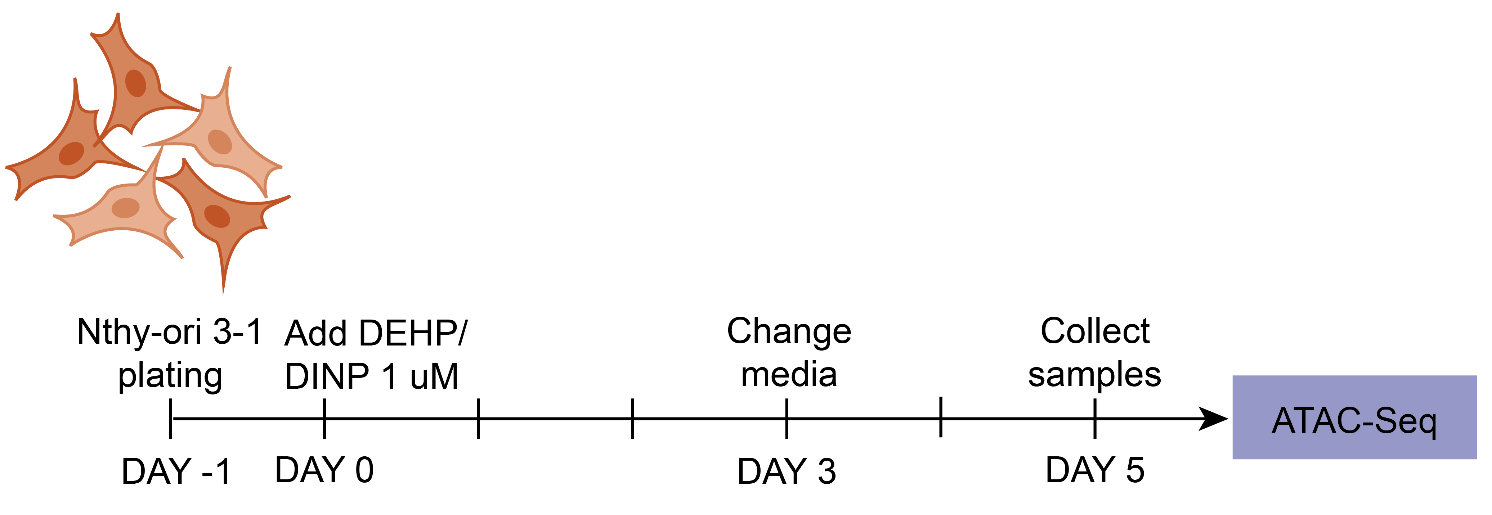
**

**Supplementary Figure 7.** Graphical representations of the exposure regimens of Nthy-ori 3-1 cells to the phthalates.


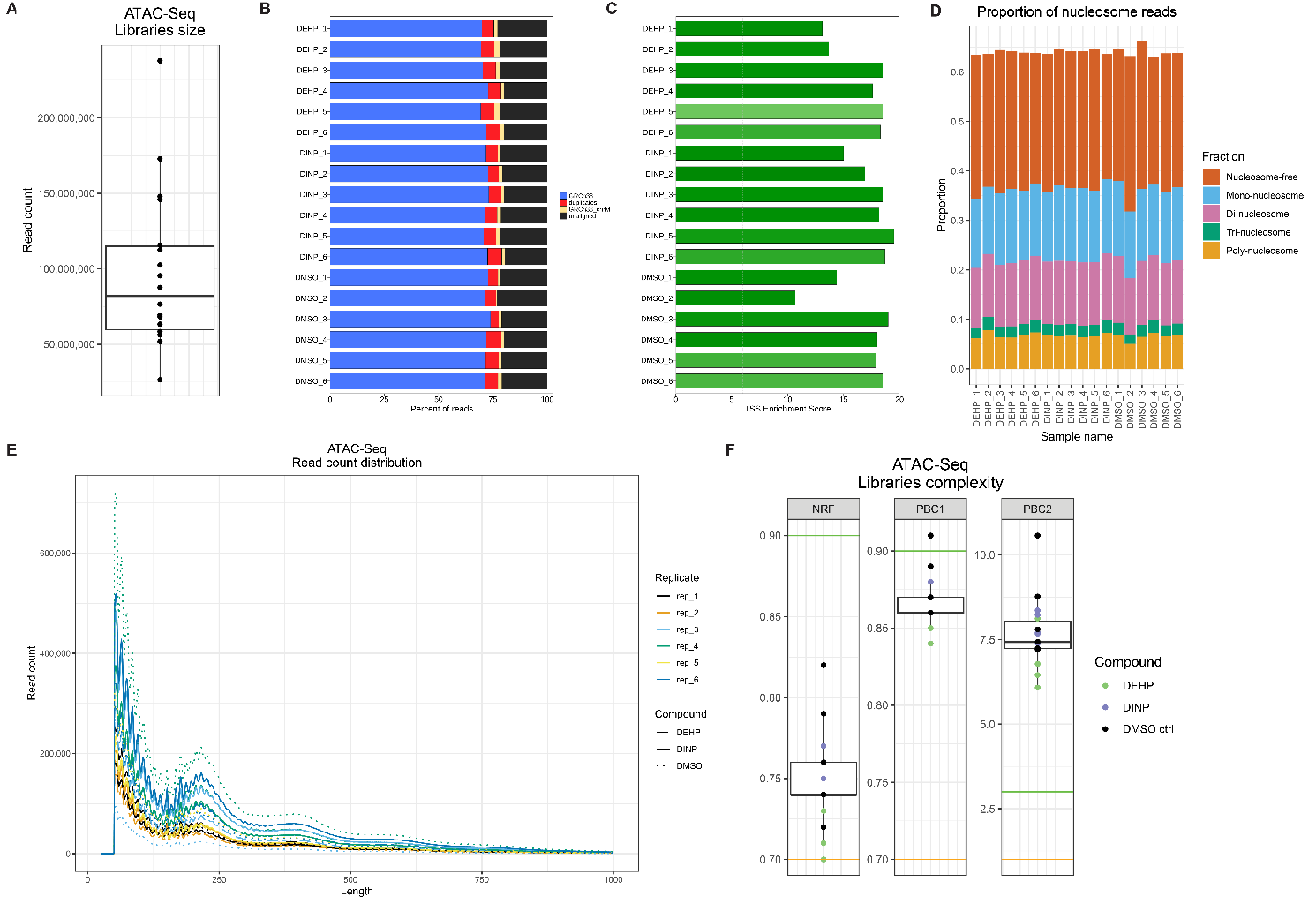


**Supplementary Figure 8.** ATAC-Seq dataset metrics. (A) Libraries size. (B) Percentage of reads aligned to GRCh38 nuclear or chromosomal genome, duplicated reads and unaligned reads. (C) Transcription Start Site (TSS) enrichment score. (D) Proportion of nucleosome-free, mono-, di-, tri- and poly-nucleosome reads. (E) Read length distribution. Each line represents a sample. (F) Boxplots of ATAC-Seq library complexity metrics. Each dot represents a sample (green = DEHP, violet = DINP, black = DMSO control). The green and orange lines represent the ENCODE thresholds for an acceptable (orange) or ideal (green) metric. (NRF = Non-Redundant Fraction; PBC1 = PCR Bottlenecking Coefficient 1; PBC2 = PCR Bottlenecking Coefficient 2). Plots (B) and (C) are output by the PEPATAC pipeline.

## Supplementary tables

Supplementary table 1. RNA-Seq samples produced in the study and the number of PCR cycles performed during Combo-Seq library preparation.

| **Same name** | **# PCR cycles** |
| --- | --- |
| DEHP_10_uM_1 | 16 |
| DEHP_10_uM_2 | 16 |
| DEHP_1_uM_1 | 16 |
| DEHP_1_uM_2 | 16 |
| DEHP_1_uM_3 | 16 |
| DEHP_100_nM_1 | 16 |
| DEHP_100_nM_2 | 14 |
| DEHP_100_nM_3 | 14 |
| DEHP_10_nM_1 | 16 |
| DEHP_10_nM_2 | 16 |
| DEHP_10_nM_3 | 16 |
| DEHP_1_nM_1 | 16 |
| DEHP_1_nM_2 | 16 |
| DEHP_1_nM_3 | 16 |
| DIDP_10_uM_1 | 16 |
| DIDP_10_uM_2 | 16 |
| DIDP_10_uM_3 | 16 |
| DIDP_1_uM_1 | 14 |
| DIDP_1_uM_2 | 14 |
| DIDP_1_uM_3 | 14 |
| DIDP_100_nM_1 | 16 |
| DIDP_100_nM_2 | 16 |
| DIDP_100_nM_3 | 16 |
| DIDP_10_nM_1 | 14 |
| DIDP_10_nM_2 | 16 |
| DIDP_10_nM_3 | 16 |
| DIDP_1_nM_1 | 16 |
| DIDP_1_nM_2 | 16 |
| DINP_10_uM_1 | 16 |
| DINP_10_uM_2 | 16 |
| DINP_10_uM_3 | 16 |
| DINP_1_uM_1 | 16 |
| DINP_1_uM_2 | 16 |
| DINP_1_uM_3 | 16 |
| DINP_100_nM_1 | 16 |
| DINP_100_nM_2 | 14 |
| DINP_100_nM_3 | 16 |
| DINP_10_nM_1 | 14 |
| DINP_10_nM_2 | 16 |
| DINP_10_nM_3 | 16 |
| DINP_1_nM_1 | 16 |
| DINP_1_nM_2 | 16 |
| DINP_1_nM_3 | 16 |
| DnOP_20_uM_1 | 16 |
| DnOP_20_uM_2 | 16 |
| DnOP_20_uM_3 | 16 |
| DnOP_2_uM_1 | 16 |
| DnOP_2_uM_2 | 16 |
| DnOP_2_uM_3 | 16 |
| DnOP_200_nM_1 | 16 |
| DnOP_200_nM_2 | 16 |
| DnOP_200_nM_3 | 14 |
| DnOP_20_nM_1 | 16 |
| DnOP_20_nM_2 | 14 |
| DnOP_20_nM_3 | 16 |
| DnOP_2_nM_1 | 16 |
| DnOP_2_nM_2 | 16 |
| DnOP_2_nM_3 | 16 |
| DMSO_ctrl_1 | 16 |
| DMSO_ ctrl_2 | 16 |
| DMSO_ ctrl_3 | 16 |
| DMSO_ ctrl_4 | 16 |
| DMSO_ ctrl_5 | 16 |
| Untr_2 | 16 |
| Untr_3 | 16 |

**Supplementary table 2.** Genes belonging to the different clusters identified by maSigPro analysis (degree=3, FDR<0.05). Note that the same cluster number in the different series does not necessarily refer to the same curve shape.

|  | **DEHP** | **DIDP** | **DINP** | **DnOP** |
| --- | --- | --- | --- | --- |
| **Cluster 1** | *Abcf3, Blmh* | *Brf2, Brip1os, Capns1, Ccdc84, Dpf2, Kank1, Kat7, Reep6, Scamp2, Tgfbi, Yars, Zswim4* | *Klf6* | *Ccnd2, Lor* |
| **Cluster 2** | *Man1a* | *Celf2, Gm8318* | *Ddr1* | *Cbx1, Ccnb1, Hspbap1, Kif23, Larp7, Lbr, Ndc80, Nfyc, Slc7a7, Srsf3, Uba2, Zzz3* |
| **Cluster 3** | *Rrp7a* | *1600014C10Rik, Dnajc8, Eda2r, Gm10323, Naa20, Zfp87* | *Dnajb1*, *Mxi1*, *Net1*, *Tbl2* | *4833411C07Rik, Gm25395, Gm7908, Lman2l, Pank4, Pcsk1n, Pi4k2a, Ptpn23, Rnasek, Slc25a34, Slc25a39, Slc38a1, Slc39a7* |
| **Cluster 4** | *Dnase1l1* | *Aplp2, Gorasp2, Ppp1r18* | *Acaa2*, *Ing5* | *Asf1b, Basp1, Gm32885, Ncoa4* |
| **Cluster 5** | *Lipt1, Serpinb9b* | *Cxcl14, Mrps34, Nop53, Prpf8, Prpsap1, Rab4a, Selenow, Slc25a3* | *Pnrc1, Setd5* | *Dap, Ddit3, Gstm1, Luc7l, Map1b, Pde4d, Slc3a2, Slc48a1, Srxn1, Tmbim1, Ttc3* |
| **Cluster 6** | *Nsd1* | *Lbp, Parm1, Sel1l, Tspan7* | *Rpl7l1* | *Gpr137b, Mid1, Nat9, Polk, Sel1l, Zbtb20* |
| **Cluster 7** | *Higd2a, Secisbp2l, Slc6a2* | *2610021A01Rik, 4930430F08Rik, Rab5c, Trmt10c, Xrcc6* | *Gm7876* | *Cyb5b, Mtres1, Sys1* |
| **Cluster 8** | *Mark4, Pkm, Ubap1* | *Cd2bp2, Ddx6, Mgme1, Snx1* | *Gm8318* | *Exoc6, Mrps22* |
| **Cluster 9** | *Cdc14a* | *Mirt1* | *Gm28661* | *Acaa2, Dnajc8* |

Supplementary table 3. Differentially accessible (DA) regions identified both in the DEHP vs DMSO and DINP vs DMSO comparisons that overlap with regulatory regions. DA regions were annotated with HOMER to identify the closest gene. A description of the gene region where the DA is located is reported. The regulatory regions that overlap with the identified DA regions were retrieved from the ENCODE Candidate Cis-Regulatory Elements (cCREs) registry (dELS = distal enhancer-like signature, pELS = proximal enhancer-like signature, PLS = promoter-like signature).

| **cCRE ID** | **cCRE type** | **Closest gene** | **Gene type** | **Gene description** | **Annotation** | **Accessibility compared to control** |
| --- | --- | --- | --- | --- | --- | --- |
| EH38D2971615 | dELS | *ASPSCR1* | Protein coding | Alveolar soft part sarcoma chromosome region, candidate 1 | Intron 3 of 14 | Increased |
| EH38D2949029 | dELS,CTCF-bound | *BCAS3* | Protein coding | Breast carcinoma amplified sequence 3 | Intron 5 of 24 | Increased |
| EH38D3590998 | dELS,CTCF-bound | *CCNI* | Protein coding | Cyclin I | TTS | Increased |
| EH38D4073480 EH38D4073481 EH38D4073482 EH38D4073483 EH38D4073484 EH38D4073485 EH38D4073486 EH38D4073487 | pELS,CTCF-bound pELS pELS,CTCF-bound pELS,CTCF-bound PLS,CTCF-bound pELS,CTCF-bound pELS pELS | *CDCA2* | Protein coding | Cell division cycle associated 2 | Promoter-TSS | Increased |
| EH38D3714835 | dELS | *CDK7* | Protein coding | Cyclin-dependent kinase 7 | Intron 11 of 11 | Increased |
| EH38D3539140 EH38D3539141 EH38D3539142 EH38D3539143 EH38D3539144 EH38D3539145 EH38D3539146 EH38D3539147 EH38D3539148 | pELS,CTCF-bound pELS,CTCF-bound pELS,CTCF-bound PLS DNase-H3K4me3 DNase-H3K4me3,CTCF-bound DNase-H3K4me3 DNase-H3K4me3 pELS | *CPLX1* | Protein coding | Complexin 1 | Promoter-TSS | Increased |
| EH38D3817447 | pELS | *DTNBP1* | Protein coding | Dystrobrevin binding protein 1 | TTS | Increased |
| EH38D3721344 | dELS | *LHFPL2* | Protein coding | Lipoma HMGIC fusion partner-like 2 | Intron 2 of 4 | Increased |
| EH38D4221907 | dELS,CTCF-bound | *FKTN* | Protein coding | Fukutin | Intron 7 of 11 | Increased |
| EH38D4071656 | dELS,CTCF-bound | *LOC100507156* | ncRNA | Uncharacterized LOC100507156 | Intron 7 of 13 | Increased |
| EH38D3794981 | dELS,CTCF-bound | *MIR8056* | ncRNA | microRNA 8056 | Intergenic | Increased |
| EH38D2141456 | pELS,CTCF-bound | *PAFAH2* | Protein coding | Platelet-activating factor acetylhydrolase 2, 40kDa | Intergenic | Increased |
| EH38D4190148 | dELS,CTCF-bound | *SLC25A51* | Protein coding | Solute carrier family 25, member 51 | Intron 5 of 5 | Increased |
| EH38D3296811 EH38D3296812 EH38D3296813 EH38D3296814 | dELS,CTCF-bound dELS,CTCF-bound dELS dELS,CTCF-bound | *SRC* | Protein coding | SRC proto-oncogene, non-receptor tyrosine kinase | Intergenic | Increased |
| EH38D3526429 | CTCF-only,CTCF-bound | *VPS8* | Protein coding | Vacuolar protein sorting 8 homolog (*S. cerevisiae*) | Intron 20 of 46 | Increased |
| EH38D3015415 | dELS | *CCBE1* | Protein coding | Collagen and calcium binding EGF domains 1 | Intron 2 of 10 | Decreased |
| EH38D3079246 | dELS,CTCF-bound | *PXDN* | Protein coding | Peroxidasin homolog (Drosophila) | Intergenic | Decreased |
| EH38D3369502 | CTCF-only,CTCF-bound | *TEX33* | Protein coding | Testis expressed 33 | Intron 5 of 5 | Decreased |
| EH38D2338583 | dELS | *TMEM72* | Protein coding | Transmembrane protein 72 | Intergenic | Decreased |

Supplementary table 4. Differentially accessible (DA) regions identified both in the DEHP vs DMSO and DINP vs DMSO comparisons that overlap with regulatory regions and fall within the Transcription Start Site (TSS) or Transcription Termination Site (TTS) of the closest gene. DA regions were annotated with HOMER to identify the closest gene. The regulatory regions that overlap with the identified DA regions were retrieved from the ENCODE Candidate Cis-Regulatory Elements (cCREs) registry (dELS = distal enhancer-like signature, pELS = proximal enhancer-like signature, PLS = promoter-like signature).

| **cCRE ID** | **cCRE type** | **Closest gene** | **Gene description** | **Annotation** | **Distance from TSS, nt** | **Accessibility compared to control** |
| --- | --- | --- | --- | --- | --- | --- |
| EH38D3590998 | dELS,CTCF-bound | *CCNI* | cyclin I | TTS | 28,721 | Increased |
| EH38D4073480 EH38D4073481 EH38D4073482 EH38D4073483 EH38D4073484 EH38D4073485 EH38D4073486 EH38D4073487 | pELS,CTCF-bound pELS pELS,CTCF-bound pELS,CTCF-bound PLS,CTCF-bound pELS,CTCF-bound pELS pELS | *CDCA2* | cell division cycle associated 2 | promoter-TSS | -221 | Increased |
| EH38D3539140 EH38D3539141 EH38D3539142 EH38D3539143 EH38D3539144 EH38D3539145 EH38D3539146 EH38D3539147 EH38D3539148 | pELS,CTCF-bound pELS,CTCF-bound pELS,CTCF-bound PLS DNase-H3K4me3 DNase-H3K4me3,CTCF-bound DNase-H3K4me3 DNase-H3K4me3 pELS | *CPLX1* | complexin 1 | promoter-TSS | -519 (DEHP vs DMSO);  -94 (DINP vs DMSO) | Increased |
| EH38D3817447 | pELS | *DTNBP1* | dystrobrevin binding protein 1 | TTS | 139,732 | Increased |
